# Supplementary material for: Combinatorial metabolic engineering using an orthogonal tri-functional CRISPR system
Source: Nat Commun. 2017 Nov 22;8:1688. doi: 10.1038/s41467-017-01695-x (PMC5700065; doi:10.1038/s41467-017-01695-x)
Supplement: Supplementary file 1 — Supplementary Information [file 41467_2017_1695_MOESM1_ESM.pdf]

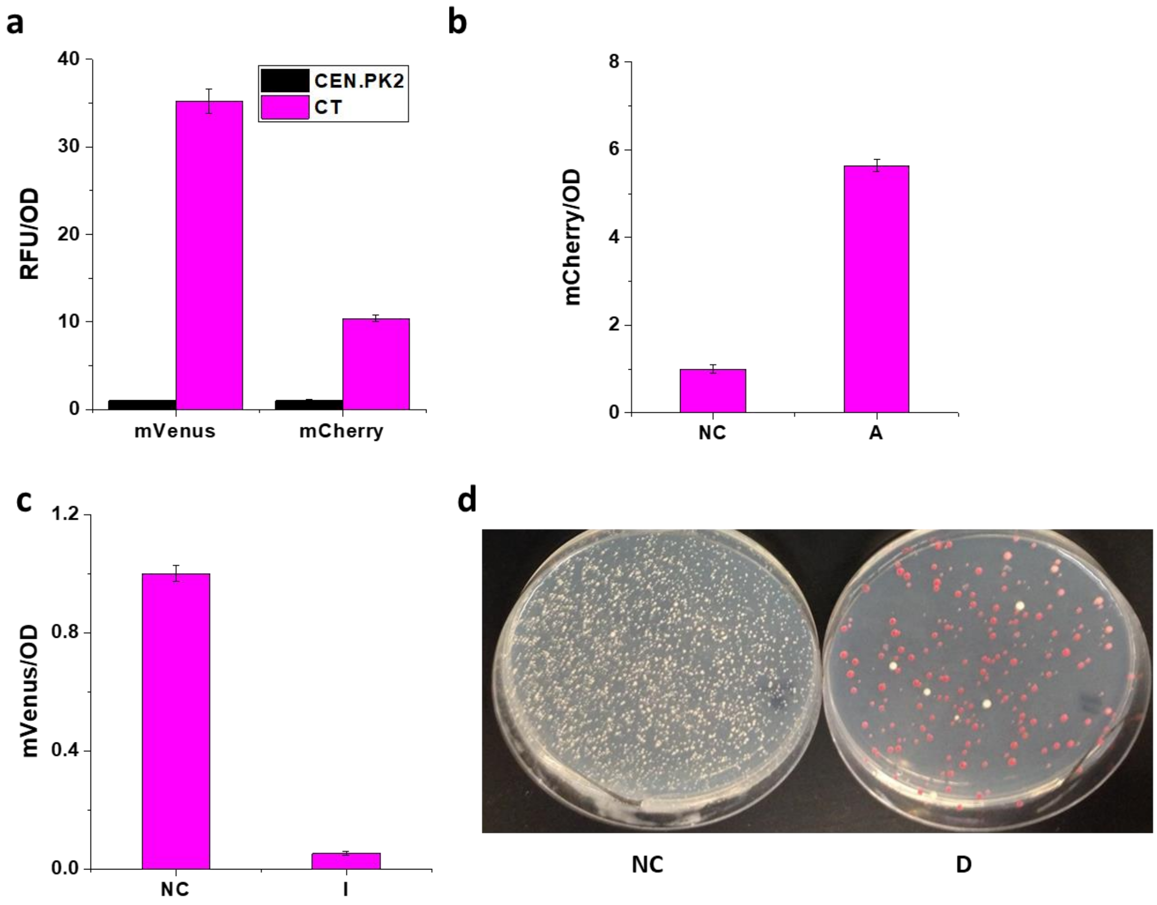

Supplementary Figure 1 | Construction of a reporter strain for CRISPR-AID. (**a**) Fluorescence intensities of mVenus and mCherry of the reporter strain. Strain CT was constructed by integrating *CYC1p-mCherry-TEF1t* and *TEF1p-mVenus-PGK1t* into the *ura3* locus of the CEN.PK2 genome. (**b**) Strain CT for CRISPRa, with dSpCad9-VPR (Sg6) for the activation of *CYC1p* included as a positive control. The expression level of *mCherry* was increased more than 5-fold. (**c**) Strain CT for CRISPRi, with dSpCad9-MXI1 (Sg1) for the interference of *TEF1p* included as a positive control. The expression level of *mVenus* was decreased around 10-fold. (**d**) Strain CT for CRISPRd, with SpCas9 (Sg11) for the deletion of *ADE2* gene included as a positive control. The deletion of *ADE2*, shown as red colonies, was achieved with an efficiency of nearly 100%. Notably, CRISPRa (**b**), CRISPRi (**c**), and CRISPRd (**d**) were carried out individually. Error bars represent the mean ± s.d. of biological quadruplicates.

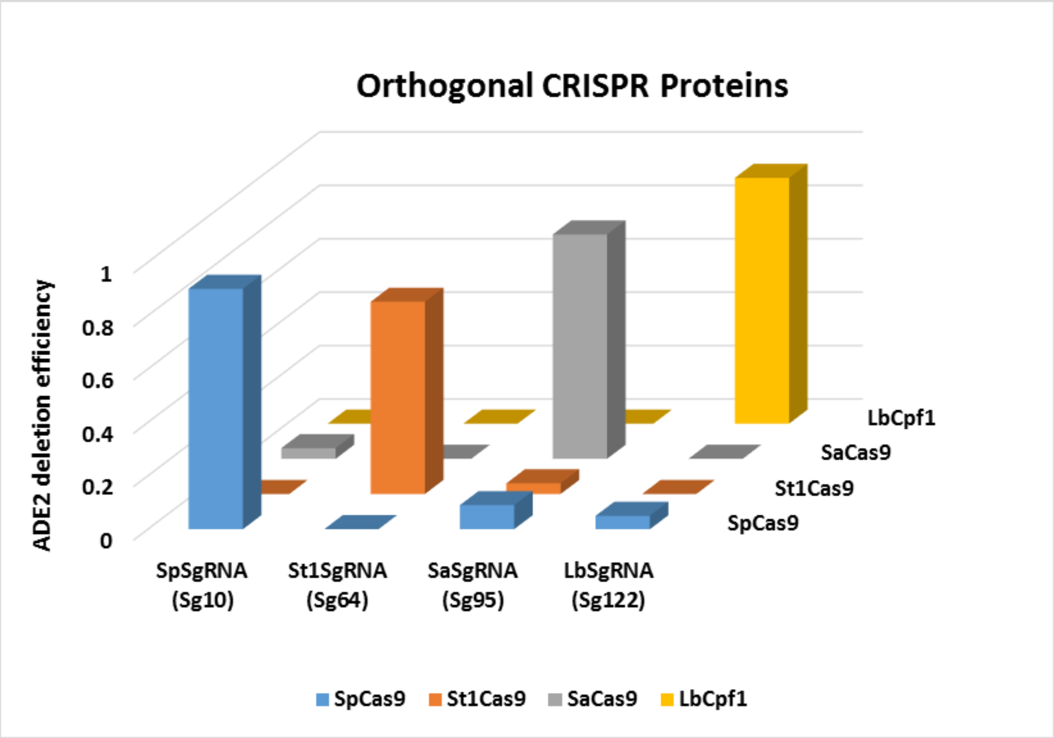

Supplementary Figure 2 | Orthogonal CRISPR proteins for CRISPR-AID. The orthogonality was tested by co-transforming the CRISPR proteins (SpCas9, St1Cas9, SaCas9, and LbCpf1) and gRNAs (Sg10, Sg64, Sg95, and Sg122) with different origins and evaluating *ADE2* deletion efficiency. In all cases, 500 ng linear donor DNA that resulted in the deletion of the whole *ADE2* coding sequences was co-transformed as well. The CRISPR proteins were only functional when their cognate gRNAs were present. 1-2 red colonies might be found on selective agar plates, but not in a reproducible manner, probably due to the spontaneous homologous recombination between the genome and the linear donor.

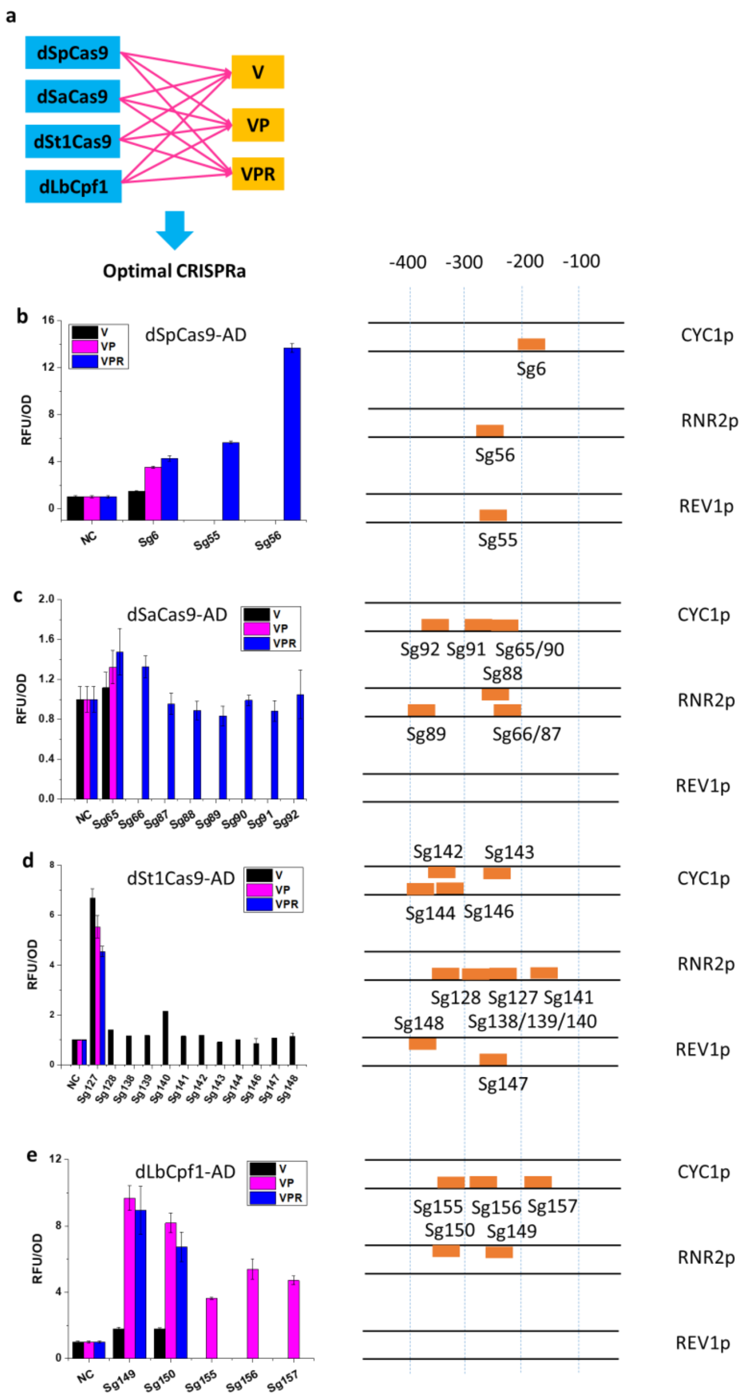

Supplementary Figure 3 | Optimization of CRISPRa. By testing all the combinations (**a**) of 4 nuclease-deficient CRISPR proteins, including dSpCas9 (**b**), dSaCas9 (**c**), dSt1Cas9 (**d**), and dLbCpf1 (**e**), and 3 activation domains (V, VP, and VPR) with different levels of strength, dSpCas9-VPR and dLbCpf1-VP were found to be the optimal combinations with the strongest activation and highest degree of flexibility in gRNA design. Error bars represent the mean ± s.d. of biological quadruplicates.

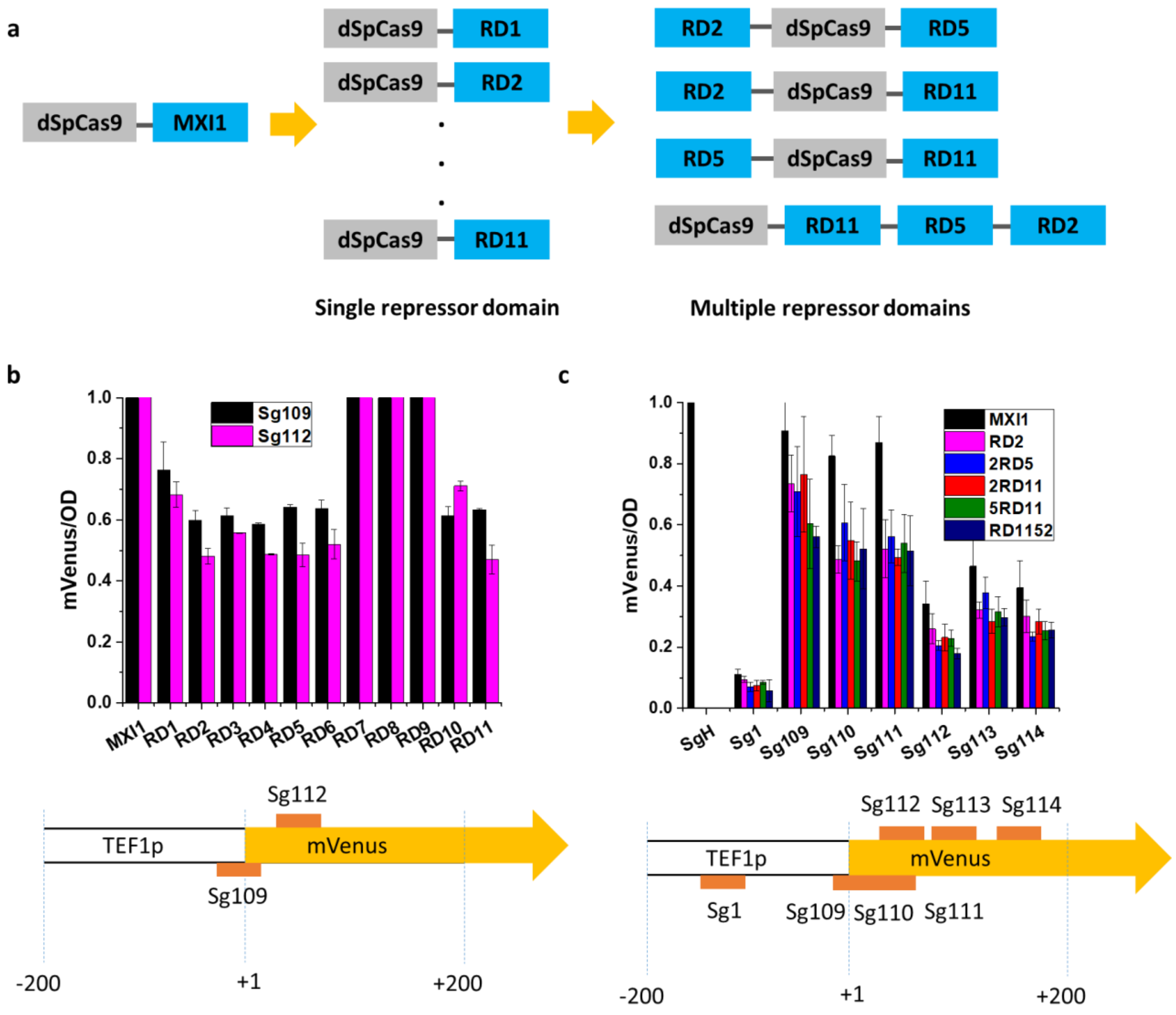

Supplementary Figure 4 | Optimization of CRISPRi by repression domain engineering. (**a**) Workflow of repression domain engineering for optimal CRISPRi. Endogenous repression domains (RD1, RD2, RD3, RD4, RD5, RD6, RD7, RD8, RD9, RD10, and RD11) were tested individually for CRISPRi efficiency and then multiple repression domains were combined either in the form of N- and C-terminal tagged (2RD5, 2RD11, and 5RD11) or tandem repeat at the C-terminus (RD1152) for maximal CRISPRi efficiency. (**b**) Enhanced CRISPRi efficiency using endogenous repression domains. The MXI1 repression domain was replaced with 11 well-characterized repression domains from *S. cerevisiae*. CRISPRi efficiency was quantified by normalizing the mVenus fluorescence intensities to those of dSpCas9-MXI1. (**c**) Further enhanced CRISPRi efficiency using multiple repression domains. The mVenus fluorescence intensities were normalized to those without gRNA targeting sequences (SgH). Error bars represent the mean ± s.d. of biological quadruplicates.

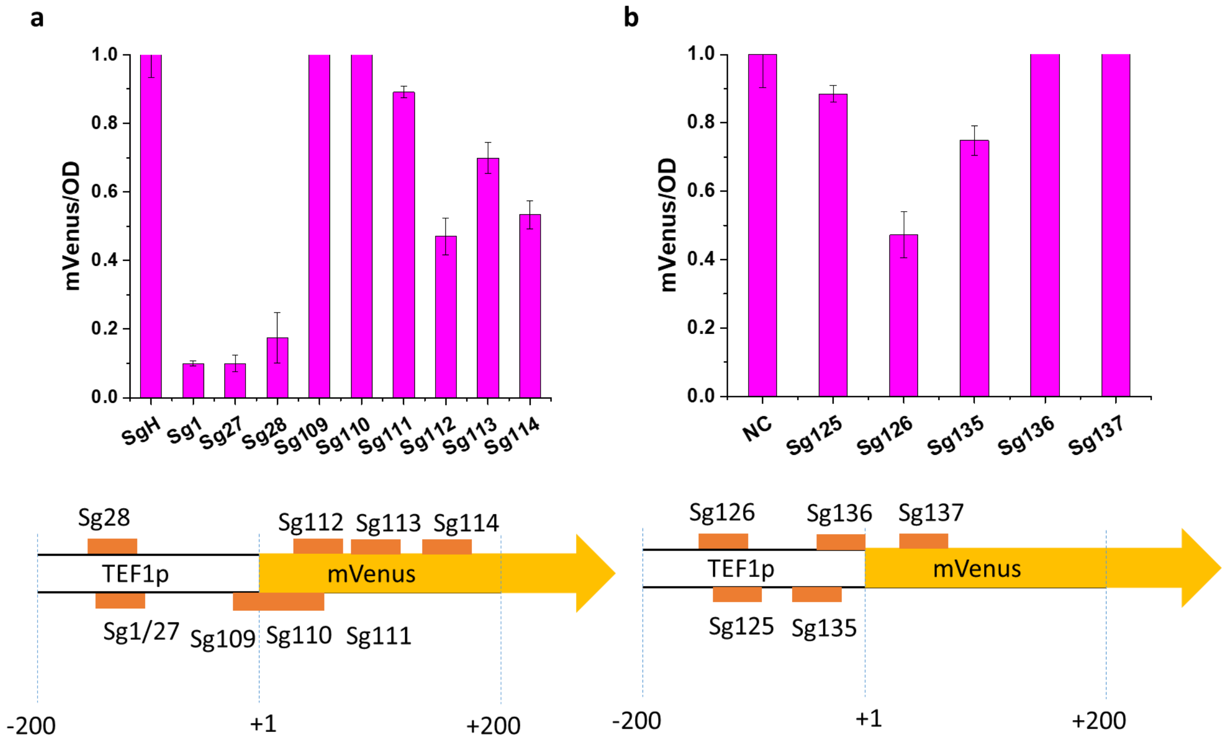

Supplementary Figure 5 | Selection of appropriate nuclease-deficient CRISPR protein for CRISPRi. The CRISPRi efficiency using dSpCas9-MXI1 (**a**) and dLbCpf1-MXI1 (**b**) were systematically compared, with several gRNAs targeting both the promoter region (blocking transcriptional initiation; Sg1, Sg27 and Sg28 for dSpCas9-MXI1; Sg125 and Sg126 for dLbCpf1-MXI1) and coding region (blocking transcriptional elongation; Sg109, Sg110, Sg111, Sg112, Sg113, and Sg114 for dSpCas9-MXI1; Sg135, Sg136, and Sg137 for dLbCpf1-MXI1) included for analysis. Generally, more efficient CRISPRi was achieved when using dSpCas9-MXI1 and targeting the promoter region. Error bars represent the mean ± s.d. of biological quadruplicates.

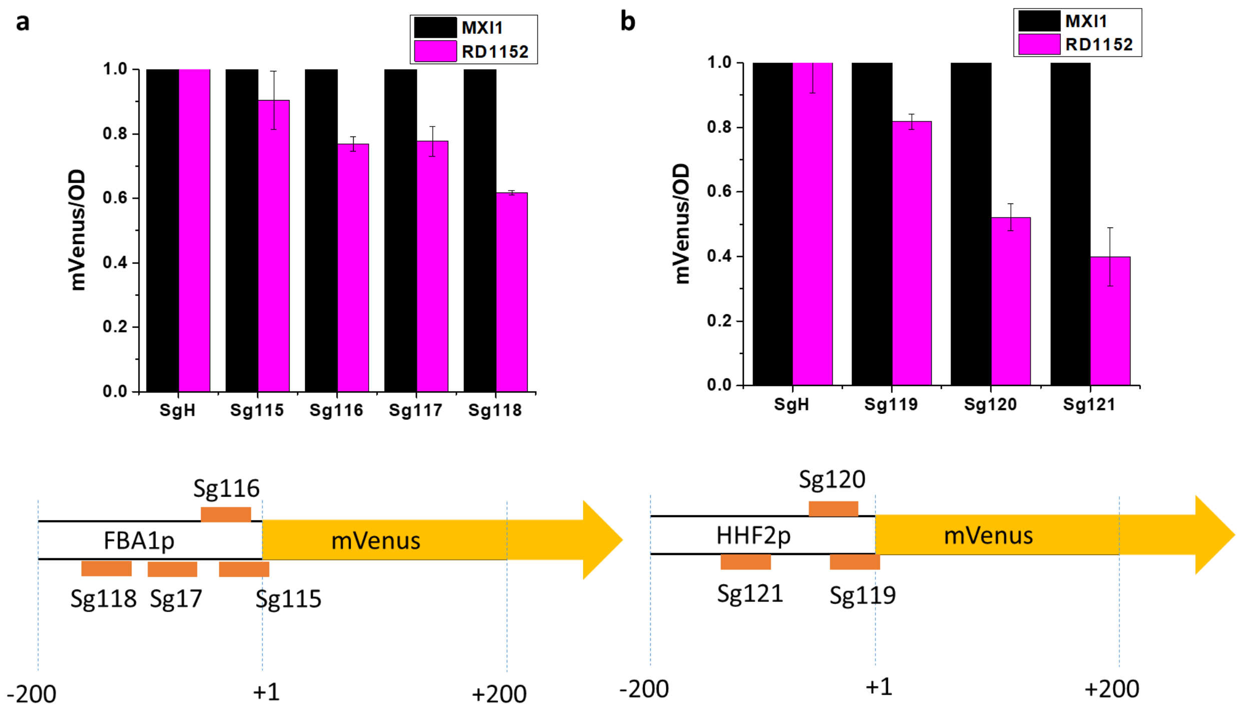

Supplementary Figure 6 | CRISPRi using the engineered repression domain for additional reporter strains. The CRISPRi efficiency using dSpCas9-MXI1 and dSpCas9-RD1152 was compared for strain CF (**a**) and strain CH (**b**), targeting *FBA1p* and *HHF2p*, respectively (**Supplementary Table S11**). The CRISPRi efficiency was normalized to that achieved using dSpCas9-MXI1. Error bars represent the mean ± s.d. of biological quadruplicates.

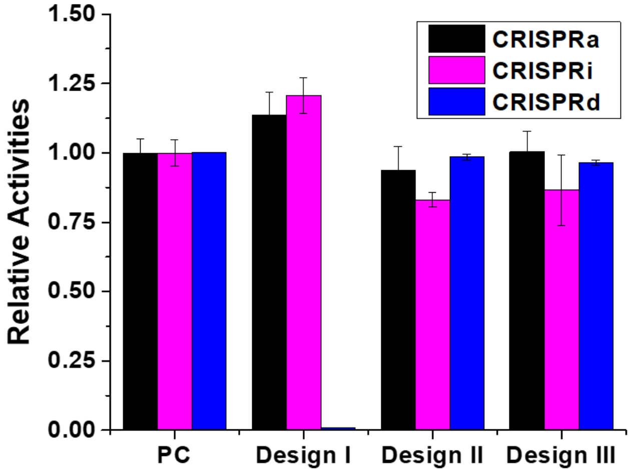

- PC: Individual gRNA cassette
- Design I: SNR52p-gRNAa-Csy4-gRNAi-Csy4-gRNAd-SUP4t
- Design II: [SNR52p-gRNAa-SUP4t]-[SNR52p-gRNAi-SUP4t]-[SNR52p-gRNAd-SUP4t]
- Design III: TEF1p-Csy4-gRNAa-Csy4-gRNAi-Csy4-gRNAd-Csy4-CY1t

Supplementary Figure 7 | Multiplex gRNA design for CRISPR-AID. Design I: expression of multiple gRNAs in a single cassette driven by a type III promoter (*SNR52p*). Design II: expression of multiple gRNAs in multiple cassettes driven by a type III promoter (*SNR52p*). Design III: expression of multiple gRNAs in a single cassette driven by a type II promoter (*TEF1p*). Plasmids containing only one gRNA cassette were included as positive controls. Design I allowed the expression of no more than 2 gRNAs. Design II and Design III allowed the expression of full length multiple gRNAs with genome engineering efficiency comparable to those with one gRNA. Error bars represent the mean ± s.d. of biological quadruplicates.

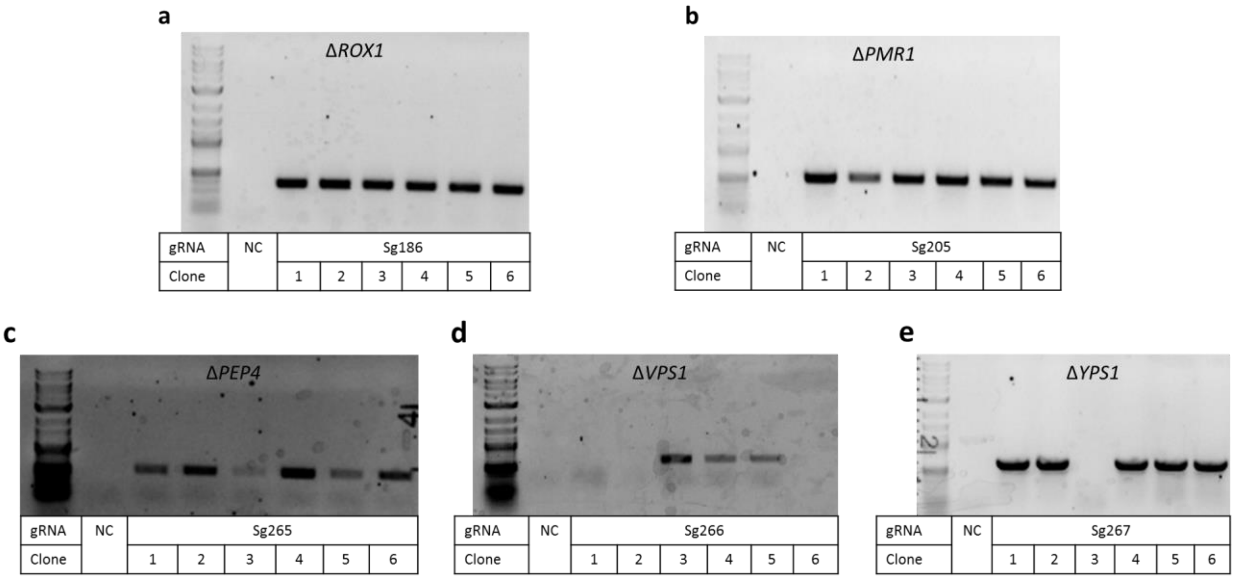

Supplementary Figure 8 | Diagnostic PCR verification of the deletion of the targeted genes by CRISPRd. After transformation of the corresponding gRNAs, single clones were picked up from the selection plates and cultured in liquid medium. Then genomic DNAs were extracted and subject to diagnostic PCR, with an amplicon only when the desired gene was disrupted.

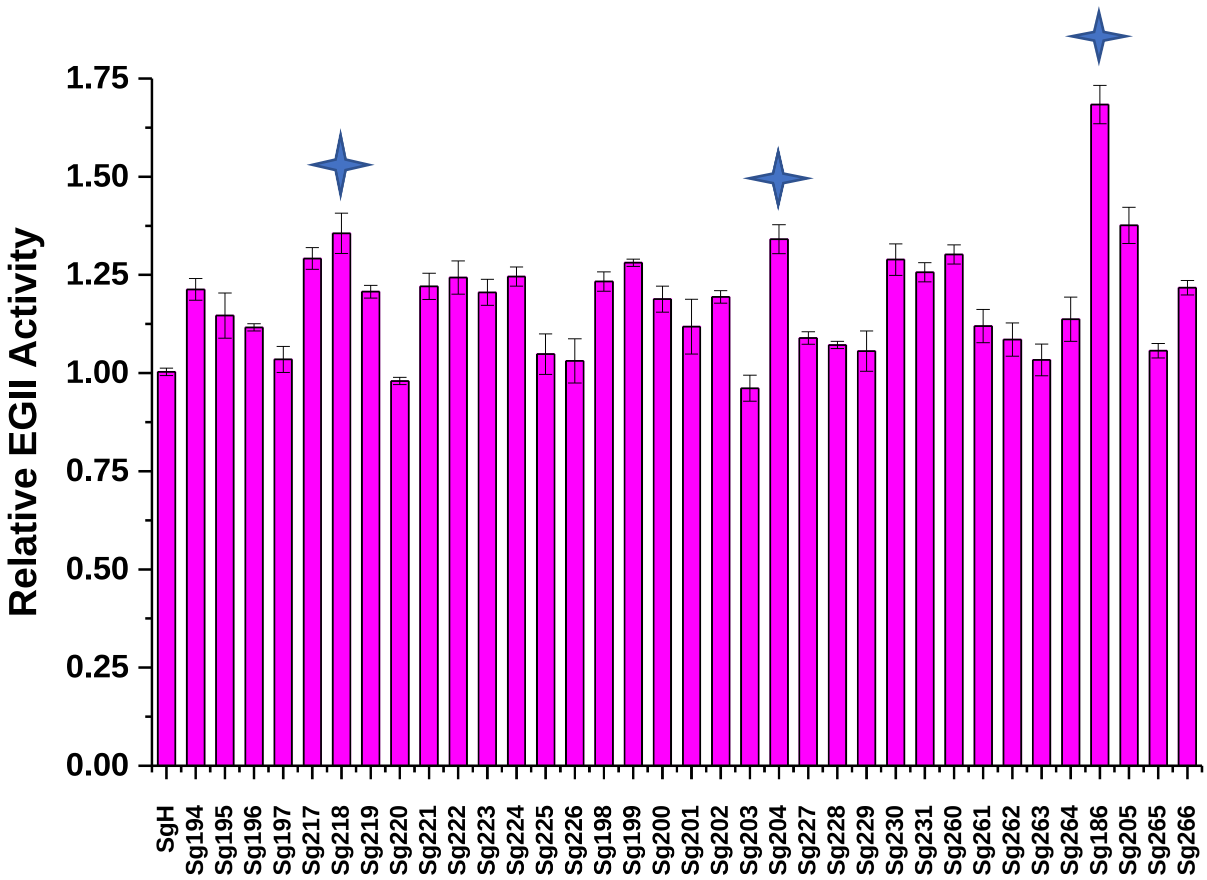

Supplementary Figure 9 | EGII activity with one gRNA. 14 CRISPRa, 17 CRISPRi, and 5 CRISPRd targets were chosen, most of which resulted in improved protein display level and EGII activity. Sg218 (*ERO2*), Sg204 (*PMR1*), and Sg186 (*ROX1*) worked the best for CRISPRa, CRISPRi, and CRISPRd, respectively. The gRNA plasmids were transformed into CEN-EGII and the resultant recombinant strains were cultured in SED-HIS-URA/G418 medium for ~3 days for cellulase activity assays. Error bars represent the mean ± s.d. of biological triplicates.

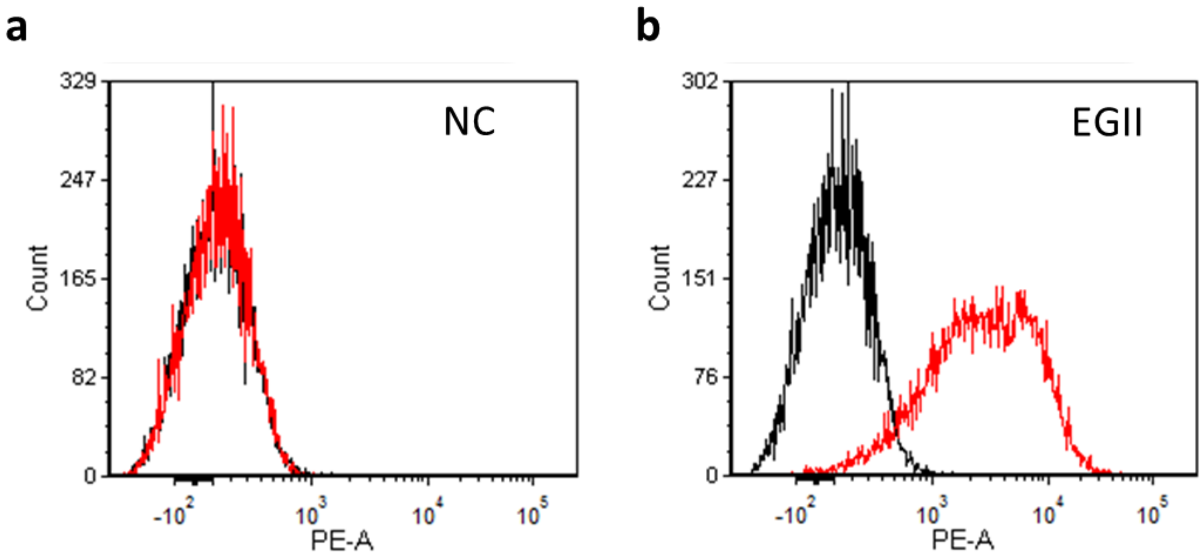

Supplementary Figure 10 | Quantification of recombinant proteins displayed on yeast surface using immunostaining. The unstained (black) and PE stained (red) control yeast strain (**a**) and EGII-displaying strain (**b**) were analyzed by flow cytometry.

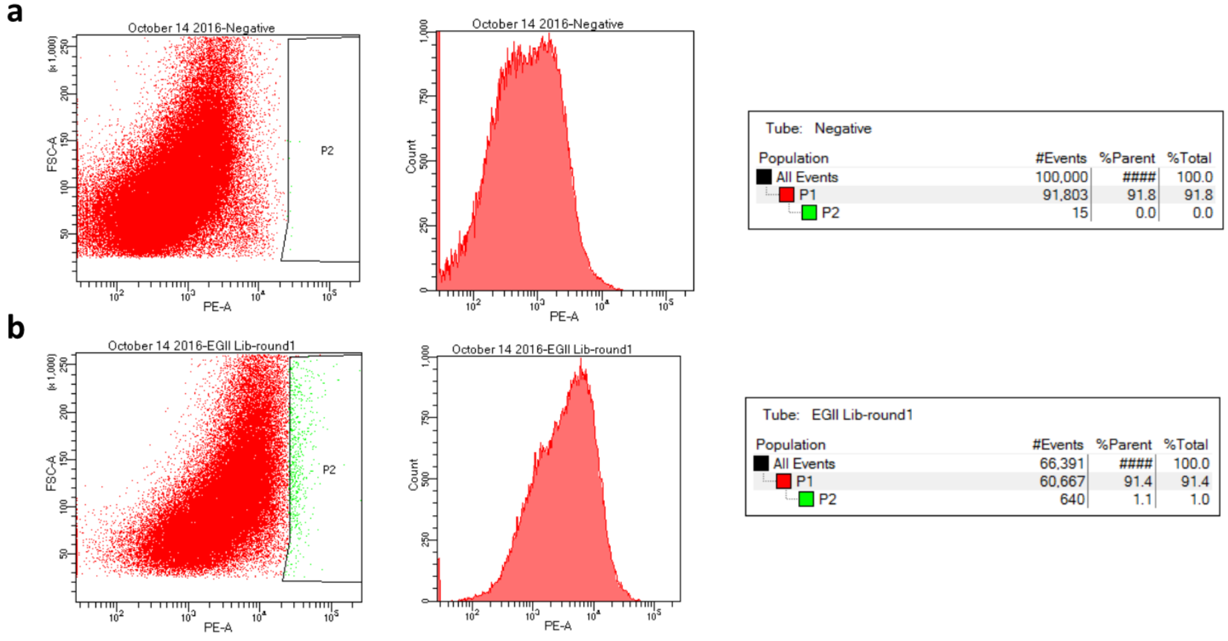

Supplementary Figure 11 | FACS sorting of the EGII-displaying library. FACS sorting profiles of the control yeast strain (**a**) and EGII-displaying library (**b**). The gate P2 was set to collect yeast cells with top 1% of the highest fluorescence.

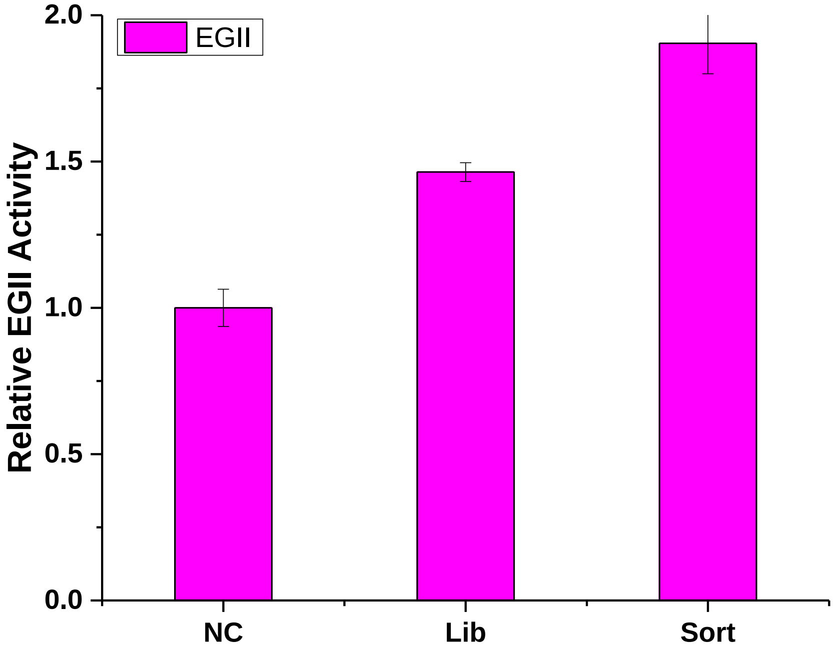

Supplementary Figure 12 | EGII activity of the transformed library and the FACS sorted library. The library strains were cultured in SED-HIS-URA/G418 medium for ~3 days for cellulase activity assays. Error bars represent the mean ± s.d. of biological triplicates.

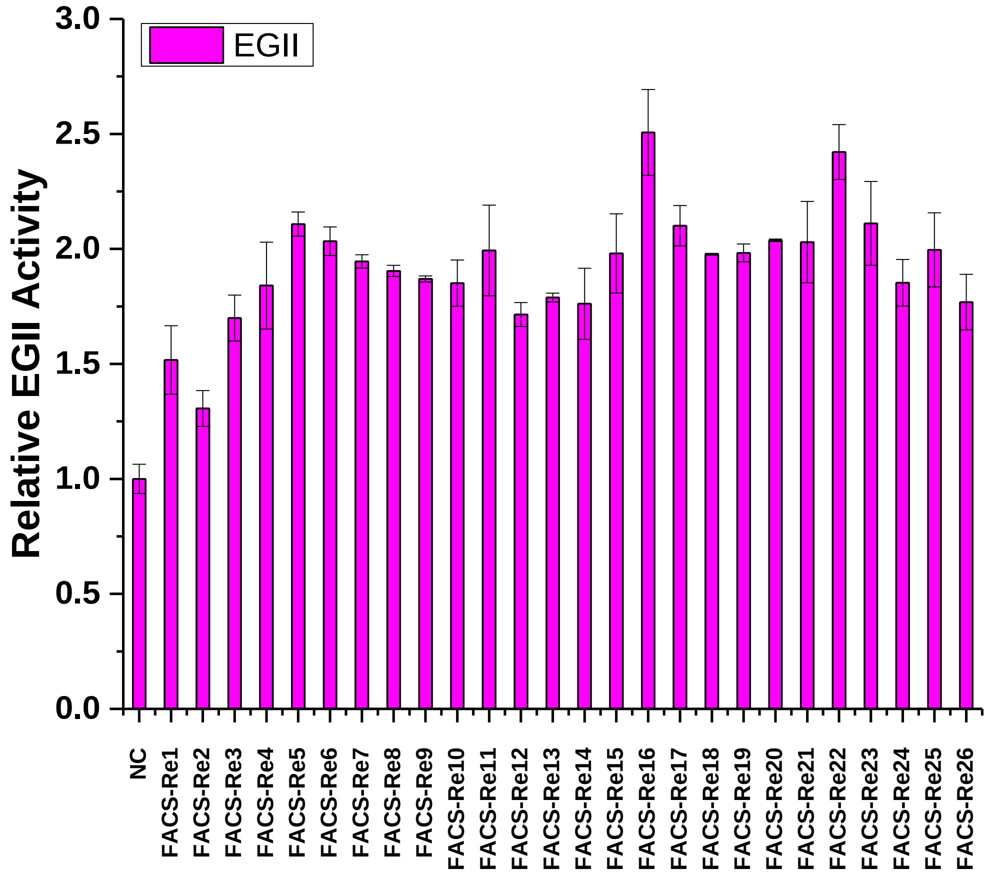

Supplementary Figure 13 | EGII activity of the FACS sorted individual clones. 96 single clones with the highest fluorescence signals were sorted using FACS, and the plasmids were extracted and re-transformed into CEN-EGII strain with a fresh background. 26 yeast strains showing the highest PE fluorescence intensity after re-transformation were chosen for cellulase activity assays. FACS-Re16 and FACS-Re22 showed the highest EGII activity. Error bars represent the mean ± s.d. of biological triplicates.

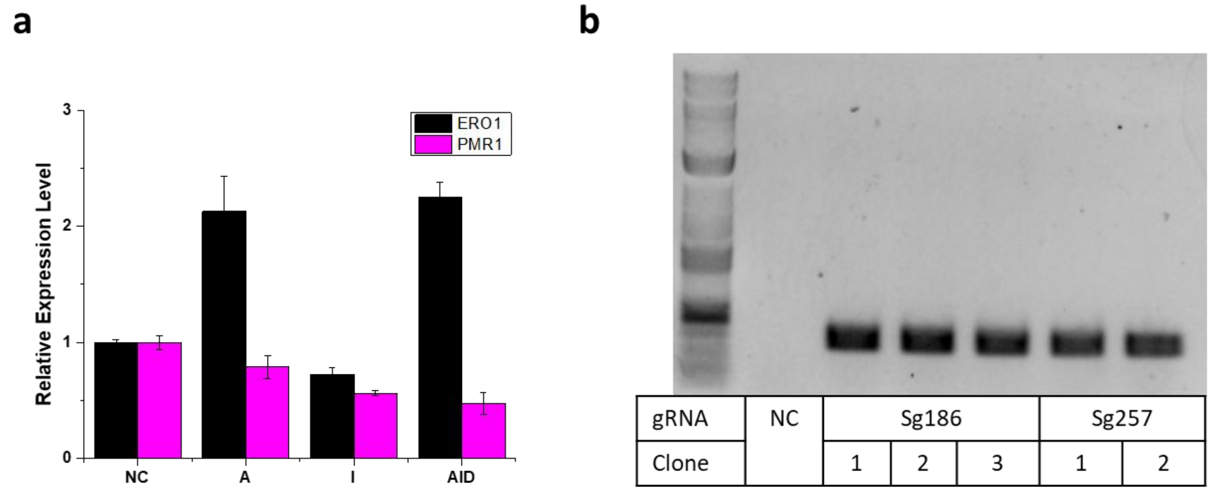

Supplementary Figure 14 | Single factor optimization using CRISPR-AID. The top candidates from each category (A-pSg218, *ERO1* activation; I-pSg204, *PMR1* interference; and D-pSg186, *ROX1* deletion) were combined (AID-pSg257) and characterized. Transcriptional regulation and genome editing were verified using qPCR and diagnostic PCR, respectively. (**a**) Verification of CRISPRa (*ERO1*) and CRISPRi (*PMR1*) for transcriptional regulation using qPCR. Error bars represent the mean ± s.d. of biological triplicates. (**b**) Verification of the disruption of *ROX1* in D (pSg186, 3 independent clones) and AID (pSg257, 2 independent clones) strains using diagnostic PCR.

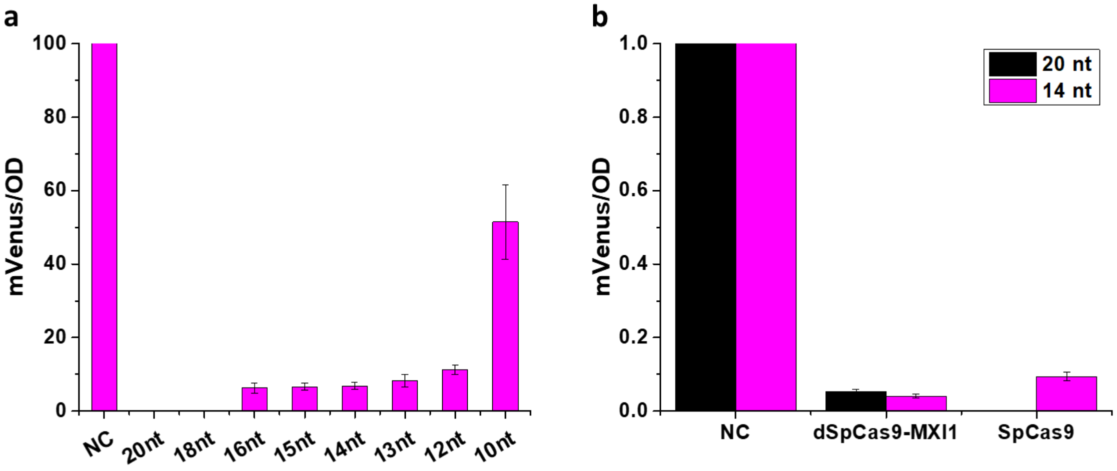

Supplementary Figure 15 | CRISPRi using truncated gRNAs. (**a**) Effect of gRNA truncation on CRISPRi efficiency. gRNAs with different length of targeting sequences were tested in catalytically active SpCas9 containing yeast strain. If the targeting sequences were longer than 16nt, no survival clones could be obtained, due to the introduction of a double strand break in the genome by the catalytically active Cas9. When the targeting sequences were between 16 and 12nt, efficient transcriptional regulation (CRISPRi in this case) could be achieved. If the targeting sequences was shorted than 12nt, CRISPRi efficiency was dramatically decreased. (**b**) A comparison of CRISPRi efficiency using full length and truncated gRNAs. The full length (Sg1) and truncated (Sg27) gRNAs were transformed into dSpCas9-MXI1 containing yeast strain and resulted in comparable CRISPRi efficiency. Error bars represent the mean ± s.d. of biological quadruplicates.

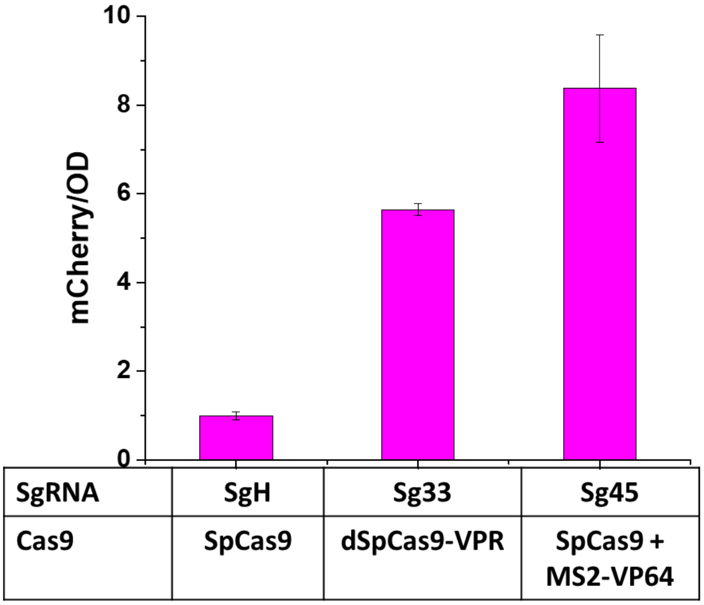

SgRNA	SgH	Sg33	Sg45
Cas9	SpCas9	dSpCas9-VPR	SpCas9 + MS2-VP64

Supplementary Figure 16 | CRISPRa using modular RNA scaffold. MS2 aptamer was included in Sg45, and the specific RNA binding protein (MS2) would recruit VP64 to activate the expression of *mCherry* under the control of *CYC1p*. CRISPRa efficiency was comparable with that achieved using dSpCas9-VPR. Error bars represent the mean ± s.d. of biological quadruplicates.

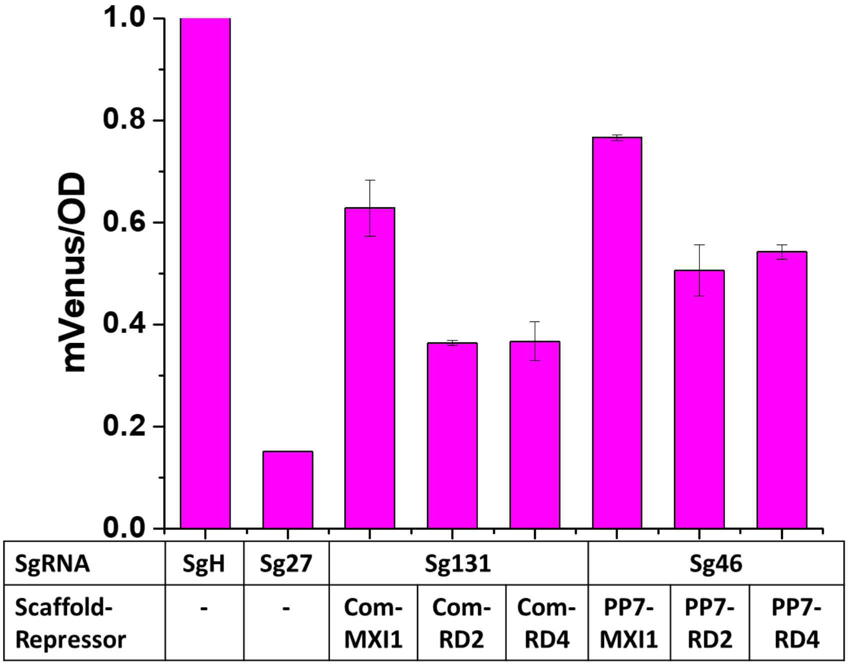

SgRNA	SgH	Sg27	Sg131			Sg46		
Scaffold-Repressor	-	-	Com-MXI1	Com-RD2	Com-RD4	PP7-MXI1	PP7-RD2	PP7-RD4

Supplementary Figure 17 | CRISPRi using engineered modular RNA scaffold. The fusion of an aptamer resulted in much lower CRISPRi efficiency, even though a repression domain was recruited through the specific RNA binding protein. The use of different aptamers and repression domains did not increase CRISPRi efficiency significantly. A much higher CRISPRi efficiency could be achieved using Sg27, without the inclusion of an aptamer and a repression domain. Notably, such high CRISPRi efficiency could only be achieved for a few cases when targeting the promoter region, if no repression domain was included. Error bars represent the mean ± s.d. of biological quadruplicates.

Supplementary Table 1 | CRISPR protein orthologues.

	PAM
SpCas9	5’-guide-NGG3’
NmCas9	5’-guide-NNNNGAAT3’
St1Cas9	5’-guide-NNAGAAW3’
SaCas9	5’-guide-NNGRRT3’
AsCpf1	5’-TTTN-guide-3’
LbCpf1	5’-TTTN-guide-3’

The gRNA structure sequences as well as the PAM sequences are different, both of which endow the activity of these CRISPR proteins to be orthogonal.

Supplementary Table 2 | Repression domains for CRISPRi in yeast.

	Repressor	Domain (aa)	Function (From SGD)
RD1	TUP1[1]	1-200	General transcription repressor that binds histones and is involved in nucleosome positioning; forms repressor complex with CYC8
RD2		73-129	
RD3		277-340	
RD4		73-340	
RD5	MIG1[2]	481-504	Transcription factor involved in glucose repression
RD6		380-504	
RD7	CRT1[3]	1-130	Major transcriptional repressor of DNA-damage-regulated genes
RD8		1-240	
RD9		709-811	
RD10	XTC1[4]	75-100	A direct transcriptional repressor
RD11	UME6[5]	508-594	Represses transcription by recruiting conserved histone deacetylase RPD3 and chromatin-remodeling factor ISW2

Well-characterized repression domains were chosen. The ideal repression domain should be small while demonstrating strong transcriptional repression.

Supplementary Table 3 | CRISPR-AID library for EGII display on yeast surface.

CRISPRa	Target	CRISPRi	Target	CRISPRd	Target
Sg194	PEX5	Sg198	SED1	Sg186	ROX1[6]
Sg195	PEX5	Sg199	SED1	Sg205	PMR1
Sg196	PTI1	Sg200	SED1	Sg265	PEP4[7]
Sg197	PTI1	Sg201	YCH1	Sg266	VPS8[7]
Sg217	CCW12[8]	Sg202	YCH1	Sg267	YPS1[9]
Sg218	ERO1[8]	Sg203	YCH1		
Sg219	HAC1[10]	Sg204	YMR1[11]		
Sg220	KAR2[12]	Sg227	OCH1[13]		
Sg221	PDI1[12]	Sg228	OCH1		
Sg222	SEC1[14]	Sg229	OCH1		
Sg223	SLY1[14]	Sg230	MNN9[13]		
Sg224	SSO1[15]	Sg231	MNN9		
Sg225	SSO2[15]	Sg260	PMR1		
Sg226	UBI4[16]	Sg261	PMR1		
		Sg262	KEX2[9]		
		Sg263	KEX2		
		Sg264	KEX2		

The empty vector without gRNA sequences was also included in the library, and a library covered all the possible combinations (15*18*6=1620) was created.

Supplementary Table 4 | Sequencing results of random clones of the combinatorial library for EGII display on yeast surface.

	A	I	D
EGII-Random 1	Sg221	Sg230	Sg265
EGII-Random 2	Sg225	Sg263	Sg205
EGII-Random 3	Sg219	Sg261	Sg267
EGII-Random 4	Sg226	Sg264	Sg205
EGII-Random 5	Sg225	Sg262	SgH
EGII-Random 6	SgH	Sg231	Sg265

Supplementary Table 5 | Sequencing results of top clones of the combinatorial library for EGII display on yeast surface.

	A	I	D
EGII-FACS5	CCW12	MNN9	PMR1
EGII-FACS11	CCW12	MNN9	PMR1
EGII-FACS16	PDI1	MNN9	PMR1
EGII-FACS17	SEC1	MNN9	PMR1
EGII-FACS22	PDI1	MNN9	PMR1
EGII-FACS23	SLY1	MNN9	PEP4

The top clones were obtained by FACS sorting of the combinatorial library and cellulase activity assay verification.

Supplementary Table 6 | Plasmids used in this study.

Plasmids	Genotype	Reference
pRS406	Integrative vector with *URA3* marker	
pH1	pRS425-PDC1p-eGFP-ADH1t	[17,18]
pH3	pRS425-ENO2p-eGFP-CYC1t-TPI1p	[17,18]
pH4	pRS425-TPI11p-eGFP-TPI1t-TEF1p	[17,18]
pH5	pRS425-TEF1p-eGFP-TEF1t	[17,18]
pH6	pRS425-TEF1t-PGK1p-BamHI-HXT7t	[17,18]
p41K-CEN-Delta	pRS-KanMX-Delta1-PmeI-CEN/ARS-PmeI-Delta2	[19]
pcDNA-NMdCas9-VPR	Harboring dNmCas9-VPR	[20]
pcDNA-SPdCas9-VPR	Harboring dSpCas9-VPR	[20]
M-ST1n-VPR	Harboring dSt1Cas9-VPR	Addgene[21]
AAV_NLS-dSaCas9-NLS-VPR	Harboring dSaCas9-VPR	Addgene[22]
pCR	Harboring SpSgRNA scaffold in *Bsa*I-free pRS423	[23]
pCT	Harboring SpCas9	[23]
pTDH3-dCas9-Mxi1	Harboring TDH3p-dSpCas9-MXI1-ADH1t	[24]
pSimpleII-U6-tracr-U6-BsmBI-NLS-NmCas9-HA-NLS(s)	Harboring NmCas9 and NmSgRNA scaffold	Addgene[25]
MSP1673	Harboring St1Cas9 and St1SgRNA scaffold	Addgene[26]
BPK2139	Harboring SaCas9	Addgene[26]
pcDNA3.1-hAsCpf1	Harboring AsCpf1	Addgene[27]
pcDNA3.1-hLbCpf1	Harboring LbCpf1	Addgene[27]
VVT1	Harboring SaSgRNA scaffold	Addgene[26]
pJZC588	SgRNA with 2x MS2 (wt+f6)	Addgene[28]
pJZC603	SgRNA with 2x PP7	Addgene[28]
pJZC620	Harboring dCas9, MCP-VP64, and PCP-VP64	Addgene[28]
YIplac211-YB/E/I	Yeast integrative vector with *URA3* marker and CrtYB, CrtE, and CrtI expression cassettes	Euroscarf[29]
YIplac128-I	Yeast integrative vector with *LEU2* marker and CrtI expression cassettes	Euroscarf[29]
p406-CT	pRS406-CYC1p-mCherry-TEF1t-TEF1p-mVenus-PGK1t	This study
p406-CF	pRS406-CYC1p-mCherry-TEF1t-FBA1p-mVenus-PGK1t	This study
p406-CH	pRS406-CYC1p-mCherry-TEF1t-HHF2p-mVenus-PGK1t	This study
p406-CR1	pRS406-CYC1p-mCherry-TEF1t-REV1p-mVenus-PGK1t	This study
p406-CR2	pRS406-CYC1p-mCherry-TEF1t-RNR2p-mVenus-PGK1t	This study
p406-YD-EGII	pRS406-TEF1p-prepro-HisTag-EGII-GS-cSAG1-PGK1t	This study
pH5-SpCas9	pRS425-TEF1p-NLS-SpCas9-NLS-TEF1t	This study
pH5-NmCas9	pRS425-TEF1p-NLS-NmCas9-NLS-TEF1t	This study
pH5-St1Cas9	pRS425-TEF1p-St1Cas9-NLS-TEF1t	This study
pH5-SaCas9	pRS425-TEF1p-SaCas9-NLS-TEF1t	This study
pH5-AsCpf1	pRS425-TEF1p-AsCpf1-NLS-TEF1t	This study
pH5-LbCpf1	pRS425-TEF1p-LbCpf1-NLS-TEF1t	This study
pSgH	pRS423*(BsaI-free)-SNR52p-BsaI-BsaI-SUP4t	This study
pSpSgH	pRS423*(BsaI-free)-SNR52p-BsaI-BsaI-SpSgRNA-SUP4t	This study
pNmSgH	pRS423*(BsaI-free)-SNR52p-BsaI-BsaI-NmSgRNA-SUP4t	This study
pSt1SgH	pRS423*(BsaI-free)-SNR52p-BsaI-BsaI-St1SgRNA-SUP4t	This study

pSaSgH	pRS423*(BsaI-free)-SNR52p-BsaI-BsaI-SaSgRNA-SUP4t	This study
pRS423-H5	pRS423-TEF1p-eGFP-TEF1t	This study
pH5-NLS-St1Cas9	pRS425-TEF1p-NLS-St1Cas9-NLS-TEF1t	This study
pH5-NLS-SaCas9	pRS425-TEF1p-NLS-SaCas9-NLS-TEF1t	This study
pH5-NLS-AsCpf1	pRS425-TEF1p-NLS-AsCpf1-NLS-TEF1t	This study
pH5-NLS-LbCpf1	pRS425-TEF1p-NLS-LbCpf1-NLS-TEF1t	This study
pTDH3-dLbCpf1-MXI1	pTDH3p-dLbCpf1-MXI1-ADH1t	This study
pTDH3-dLbCpf1-V	pTDH3p-dLbCpf1-V-ADH1t	This study
pTDH3-dLbCpf1-VP	pTDH3p-dLbCpf1-VP-ADH1t	This study
pTDH3-dLbCpf1-VPR	pTDH3p-dLbCpf1-VPR-ADH1t	This study
pH6-dSpCas9-V	pRS425-PGK1p-dSpCas9-V-HXT7t	This study
pH6-dSpCas9-VP	pRS425-PGK1p-dSpCas9-VP-HXT7t	This study
pH6-dSpCas9-VPR	pRS425-PGK1p-dSpCas9-VPR-HXT7t	This study
pH6-dSt1Cas9-V	pRS425-PGK1p-dSt1Cas9-V-HXT7t	This study
pH6-dSt1Cas9-VP	pRS425-PGK1p-dSt1Cas9-VP-HXT7t	This study
pH6-dSt1Cas9-VPR	pRS425-PGK1p-dSt1Cas9-VPR-HXT7t	This study
pH6-dSaCas9-V	pRS425-PGK1p-dSaCas9-V-HXT7t	This study
pH6-dSaCas9-VP	pRS425-PGK1p-dSaCas9-VP-HXT7t	This study
pH6-dSaCas9-VPR	pRS425-PGK1p-dSaCas9-VPR-HXT7t	This study
pTDH3-dSpCas9-RD1	pTDH3p-dSpCas9-RD1-ADH1t	This study
pTDH3-dSpCas9-RD2	pTDH3p-dSpCas9-RD2-ADH1t	This study
pTDH3-dSpCas9-RD3	pTDH3p-dSpCas9-RD3-ADH1t	This study
pTDH3-dSpCas9-RD4	pTDH3p-dSpCas9-RD4-ADH1t	This study
pTDH3-dSpCas9-RD5	pTDH3p-dSpCas9-RD5-ADH1t	This study
pTDH3-dSpCas9-RD6	pTDH3p-dSpCas9-RD6-ADH1t	This study
pTDH3-dSpCas9-RD7	pTDH3p-dSpCas9-RD7-ADH1t	This study
pTDH3-dSpCas9-RD8	pTDH3p-dSpCas9-RD8-ADH1t	This study
pTDH3-dSpCas9-RD9	pTDH3p-dSpCas9-RD9-ADH1t	This study
pTDH3-dSpCas9-RD10	pTDH3p-dSpCas9-RD10-ADH1t	This study
pTDH3-dSpCas9-RD11	pTDH3p-dSpCas9-RD11-ADH1t	This study
pTDH3-RD2-dSpCas9-RD5	pTDH3p-RD2-dSpCas9-RD5-ADH1t	This study
pTDH3-RD2-dSpCas9-RD11	pTDH3p-RD2-dSpCas9-RD11-ADH1t	This study
pTDH3-RD5-dSpCas9-RD11	pTDH3p-RD5-dSpCas9-RD11-ADH1t	This study
pTDH3-dSpCas9-RD1152	pTDH3p-dSpCas9-RD11-RD5-RD2-ADH1t	This study
pH4-dSpCas9-RD1152	pRS425-TPI1p-dSpCas9-RD11-RD5-RD2-TPI1t-TEF1p	This study
pH3-Csy4	pRS425-ENO2p-Csy4-PGK1t-TPI1p	This study
pAID6	p41K-CEN-Delta-TDH3p-dLbCpf1-VP-ADH1t-ENO2p-Csy4-PGK1t-TPI1p-dSpCas9-RD11-RD5-RD2-TPI1t-TEF1p-SaCas9-NLS-TEF1t	This study
pSpMS2SgH	pRS423*-SNR52p-BsaI-BsaI-SpSgRNA-MS2-SUP4t	This study
pSpPP7SgH	pRS423*-SNR52p-BsaI-BsaI-SpSgRNA-PP7-SUP4t	This study
pSpComSgH	pRS423*-SNR52p-BsaI-BsaI-SpSgRNA-Com-SUP4t	This study
pH1-PP7-MXI1	pRS425-PDC1p-PP7-MXI1-ADH1t	This study
pH1-PP7-RD2	pRS425-PDC1p-PP7-RD2-ADH1t	This study
pH1-PP7-RD4	pRS425-PDC1p-PP7-RD4-ADH1t	This study

pH1-Com-MXI1	pRS425-PDC1p-Com-MXI1-ADH1t	This study
pH1-Com-RD2	pRS425-PDC1p-Com-RD2-ADH1t	This study
pH1-Com-RD4	pRS425-PDC1p-Com-RD4-ADH1t	This study
pH4-MS2-VP64	pRS425-TPI1p-MS2-VP64-TPI1t-TEF1p	This study

Supplementary Table 7 | gRNA plasmids constructed in this study.

Plasmid	Cas9	Target	AID	Position	Strand	Protospacer	PAM
pSg1	Sp	TEF1p	i	-115 to -134	t	ttgatatttaagttattaaa	tgg
pSg6	Sp	CYC1p	a	-183 to -202	t	actttagtgctgacacatac	agg
pSg10	Sp	ADE2	d	157 to 177	nt	gatatcaagaggattggaaa	agg
pSg11	Sp	Same as psg10, except that 100 bp hr donor was integrated (HI-CRISPR)					
pSg12	Nm	ADE2	d	394 tp 413	t	acgtccctattgaatgttgg	aagagatt
pSg13	Nm	ADE2	d	826 to 845	t	aactctggacattataccat	tgatgctt
pSg14	St1	ADE2	d	548 to 567	t	aaaaatgggcaccatttact	aaagaat
pSg15	St1	ADE2	d	622 to 641	t	ccaattgtagagactatcca	caagga
pSg27	Sp	TEF1p	i	-115 to -128	t	tttaagttattaaa	tgg
pSg28	Sp	TEF1p	i	-125 to -138	nt	taaatatcaatggg	agg
pSg29	Nm	ADE2	d	871 to 890	t	gaagctcatttgagatcaat	attggatt
pSg30	St1	ADE2	d	466 to 485	t	ggaagaggtaacttcgttgt	aaagaat
pSg31	Sa	ADE2	d	833 to 855	nt	gcaagcatcaatggtataatgtc	cagagt
pSg32	Nm	ADE2	d	473 to 496	t	gtaacttcgttgtaaagaataagg	aaatgatt
pSg33	Sp	CYC1p	a	-183 to -196	t	gtgctgacacatac	agg
pSg35	Sp	TEF1p	i	-115 to -134	t	gatatttaagttattaaa	tgg
pSg36	Sp	TEF1p	i	-115 to -134	t	tatttaagttattaaa	tgg
pSg37	Sp	TEF1p	i	-115 to -134	t	atttaagttattaaa	tgg
pSg38	Sp	TEF1p	i	-115 to -134	t	ttaagttattaaa	tgg
pSg39	Sp	TEF1p	i	-115 to -134	t	taagttattaaa	tgg
pSg40	Sp	TEF1p	i	-115 to -134	t	agttattaaa	tgg
pSg45	SpMS2	CYC1p	a	The same as Sg33			
pSg46	SpPP7	TEF1p	i	The same as Sg27			
pSg55	Sp	REV1p	a	-250 to -269	t	gaaaaaagtagcta	agg
pSg56	Sp	RNR2p	a	-242 to -261	t	ccgtaccataccct	tgg
pSg64	St1	ADE2	d	621 to 640	nt	ggatagtctctacaattggg	taagaaa
pSg65	Sa	CYC1p	a	-217 to -239	t	tccgccaggcgtgtatatatagc	gtggat
pSg66	Sa	RNR2p	a	-203 to -225	t	aacgaagcaggaaatgagagaat	gagagt
pSg68	As	ADE2	d	155 to 177	nt	gatatcaagaggattggaaaagg	tttc
pSg69	Lb	ADE2	d	155 to 177	nt	gatatcaagaggattggaaaagg	tttc
pSg87	Sa	RNR2p	a	-203 to -223	t	cgaagcaggaaatgagagaat	gagagt
pSg88	Sa	RNR2p	a	-219 to -239	nt	cttcgttcatttcgagtttcc	aagggt
pSg89	Sa	RNR2p	a	-384 to -404	t	cagacctccctgcgagcgggc	atgggt
pSg90	Sa	CYC1p	a	-217 to -237	t	cgccaggcgtgtatatatagc	gtggat
pSg91	Sa	CYC1p	a	-277 to -297	t	tcatttggcgagcgttggttg	gtggat
pSg92	Sa	CYC1p	a	-337 to -357	t	gatctttccggtctctttggc	gtggat
pSg93	Sa	ADE2d	d	367 to 387	nt	ggcttgttccacaggaacact	ttgggt
pSg94	Sa	ADE2d	d	438 to 458	nt	gccaaagtcctcgacttcaag	acgaat
pSg95	Sa	ADE2d	d	695 to 715	nt	acaacttcgccttaagttgaa	cggagt

pSg109	Sp	TEF1p	i	1 to -19	t	tctaagttttaattacaaaa	tgg
pSg110	Sp	mVenus	i	3 to 22	t	ggaattcgtgagcaagggcg	tgg
pSg111	Sp	mVenus	i	21 to 40	t	cgaggagctgttcaccgggg	cgg
pSg112	Sp	mVenus	i	38 to 57	nt	gaccaggatgggcaccaccc	agg
pSg113	Sp	mVenus	i	54 to 73	nt	cgtcgccgtccagctcgacc	ggg
pSg114	Sp	mVenus	i	140 to 159	nt	ggtggtgcagatcagcttca	tgg
pSg115	Sp	FBA1p	i	1 to -19	t	caagtaatacatattcaaaa	tgg
pSg116	Sp	FBA1p	i	-4 to -23	nt	gaatatgtattacttggtta	tgg
pSg117	Sp	FBA1p	i	-48 to -67	t	aagaacagaagaataacgca	agg
pSg118	Sp	FBA1p	i	-145 to -164	t	ttatccctcatgttgtctaa	cgg
pSg119	Sp	HHF2p	i	1 to -19	t	caatcaatacaataaaataa	tgg
pSg120	Sp	HHF2p	i	-29 to -48	nt	tactcttttgaacaagatgt	agg
pSg121	Sp	HHF2p	i	-107 to -120	t	ataagtatattaggatgagg	cgg
pSg122	Lb	ADE2	d	219 to 241	nt	gtgtaggaacatcaacatgctca	ttta
pSg123	Lb	ADE2	d	282 to 304	t	cccttctccagaaacaatcagat	ttta
pSg124	Lb	ADE2	d	430 to 452	t	ccattcgtcttgaagtcgaggac	tttt
pSg125	Lb	TEF1	i	-101 to -123	t	agttattaaatggtcttcaattt	ttta
pSg126	Lb	TEF1	i	-118 to -140	nt	ataacttaaatatcaatgggagg	ttta
pSg127	St1	RNR2	a	-210 to -229	t	aatgaacgaagcaggaaatg	agagaat
pSg128	St1	RNR2	a	-308 to -327	t	gcgtgttgttgctgctgaca	aaagaaa
pSg131	SpCom	TEF1	i	The same as Sg27			
pSg135	Lb	TEF1	i	-33 to -55	t	cttcttgctcattagaaagaaag	ttta
pSg136	Lb	TEF1	i	-5 to -27	nt	taattaaaacttagattagattg	tttg
pSg137	Lb	mVenus	i	51 to 73	nt	cgtcgccgtccagctcgaccagg	ttta
pSg138	St1	RNR2	a	-277 to -296	t	tttcttagcaaagcaaagga	ggggaa
pSg139	St1	RNR2	a	-220 to -239	t	ggaaactcgaaatgaacgaa	gcagga
pSg140	St1	RNR2	a	-274 to -293	t	cttagcaaagcaaaggaggg	gaagca
pSg141	St1	RNR2	a	-164 to -183	t	atagcggtagtgtttgcgcg	ttacca
pSg142	St1	CYC1	a	-327 to -346	nt	gtaaaccccggccaaagaga	ccggaa
pSg143	St1	CYC1	a	-226 to -245	nt	acacgcctggcggatctgct	cgagga
pSg144	St1	CYC1	a	-383 to -402	t	acctgaatctaaaattcccg	ggagca
pSg145	Sa	ADE2	d	The same as Sg95, but with 100 bp HR (HI-CRISPR)			
pSg146	St1	CYC1	a	-319 to -338	t	gccggggtttacggacgatg	gcagaa
pSg147	St1	REV1	a	-247 to -266	t	gacggaaaaaagtagctaag	gaagaa
pSg148	St1	REV1	a	-383 to -402	nt	caaagcattcaattcaaatg	aaagaa
pSg149	Lb	RNR2	a	-239 to -261	nt	caagggtatggtacggtgctatc	tttc
pSg150	Lb	RNR2	a	-309 to -331	nt	tcagcagcaacaacacgctacgc	tttg
pSg155	Lb	CYC1	a	-306 to -328	t	cggacgatggcagaagaccaaag	ttta
pSg156	Lb	CYC1	a	-269 to -291	t	gcgagcgttggttggtggatcaa	tttg
pSg157	Lb	CYC1	a	-174 to -196	t	gtgctgacacatacaggcatata	ttta
pSg163	AID6	Sg156-Sg112-Sg145					

pSg172	Sp	ERG9	i	-87 to -106	t	ataaatggaaagttaggaca	ggg
pSg175	Lb	HMG1	a	-228 to -250	t	cggctatgaaaagctgttgttcg	tttt
pSg186	Sa	ROX1	d	68 to 88	t	actaccacaggatcttaatag	acgaat
pSg194	Lb	PEX5	a	-182 to -204	nt	catattcgaagcttacaatcgag	ttta
pSg195	Lb	PEX5	a	-296 to -318	t	taccagcaatcagctgactaaca	ttta
pSg196	Lb	PTI1	a	-259 to -281	t	ttgctcttacccgactctgaaga	ttta
pSg197	Lb	PTI1	a	-174 to -196	nt	gcaagacctcaaacaatcgtact	tttc
pSg198	Sp	SED1	i	-165 to -187	t	gctggggtagaactagagta	agg
pSg199	Sp	SED1	i	-127 to -146	nt	ttatatgacagttcaaaaga	ggg
pSg200	Sp	SED1	i	101 to 120	nt	ggaagtggagatggaagagg	agg
pSg201	Sp	YCH1	i	-169 to -188	t	ctacatgcaaacgacaaata	cgg
pSg202	Sp	YCH1	i	-61 to -80	nt	gctgaaaactgtatgtgcgg	agg
pSg203	Sp	YCH1	i	43 to 62	nt	atccaacgatgcaattcagt	cgg
pSg204	Sp	PMR1	i	-107 to -126	nt	aaatgggaatggaaagaacg	ggg
pSg205	Sa	PMR1	d	683 to 703	nt	atctctcagaaatcggtacaa	ttgaat
pSg217	Lb	CCW12	a	-242 to -264	t	caacaactatctgcgataactca	tttg
pSg218	Lb	ERO1	a	-221 to -243	nt	cagggtcttctataagagaaacc	tttc
pSg219	Lb	HAC1	a	-266 to -288	nt	agccctacttaatgctgagccac	tttt
pSg220	Lb	KAR2	a	-214 to -236	t	gctatgttagctgcaactttcta	tttt
pSg221	Lb	PDI1	a	-275 to -297	t	gaaacacgtgtcctgaaaattat	tttc
pSg222	Lb	SEC1	a	-235 to -257	t	aaaatcatcgaatagccgatcga	ttta
pSg223	Lb	SLY1	a	-217 to -239	t	ccagtcactatcatcatcatcat	tttt
pSg224	Lb	SSO1	a	-256 to -278	nt	acgggcaaaaactggattctccc	ttta
pSg225	Lb	SSO2	a	-234 to -256	t	tgtcttacgagccgggtaccaag	ttta
pSg226	Lb	UBI4	a	-231 to -253	t	caggggcgatgccacttatcagt	tttt
pSg227	Sp	OCH1	i	-134 to -153	nt	ggattggcgagaaataatgt	cgg
pSg228	Sp	OCH1	i	-113 to -132	nt	gcagatggggagagagaatg	tgg
pSg229	Sp	OCH1	i	20 to 39	nt	tttccttgtagcgatcaggt	ggg
pSg230	SP	MNN9	i	-112 to -131	nt	gaaataacgggtcccaagag	cgg
pSg231	Sp	MNN9	i	27 to 46	nt	cccacgggttctttcttagg	cgg
pSg239	AID	Sg175-Sg172-Sg186					
pSg257	AID	Sg218-Sg204-Sg186					
pSg260	Sp	PMR1	i	-129 to -148	nt	gcgagcaaacactattatga	tgg
pSg261	Sp	PMR1	i	86 to 105	nt	agaagggcttggtttcgaaa	ggg
pSg262	Sp	KEX2	i	-116 to -135	nt	caaaacgggatatttaagcc	agg
pSg263	Sp	KEX2	i	-76 to -95	nt	agccgaatgaatgaaatatg	tgg
pSg264	Sp	KEX2	i	56 to 75	nt	ttgttgtgatgatacaagag	cgg
pSg265	Sa	PEP4	d	821 to 841	t	ttgaaggtatcggtttaggcg	acgagt
pSg266	Sa	VPS8	d	470 to 490	t	tatgcatttggaacttgaacg	tagggt
pSg267	Sa	YPS1	d	1190 to 1210	nt	atacgtaataccctatcctgg	aagagt
FACS16	AID	Sg221-Sg230-Sg205 (the same as FACS22)					

pSg417	AI	Sg221-Sg230-SgH
pSg418	AD	Sg221-SgH-Sg205
pSg419	ID	SgH-Sg230-Sg205
pSg585	AI	Sg175-Sg172-SgH

Supplementary Table 8 | Oligonucleotides used in this study.

Oligos	Sequences (5'-3')	Applications
CT-F1	ctcactatagggcgaattgggtaccctcgagaattttttggaaaaccaag	Construct p406-CT (Gibson)
CT-R1	gttatcctcctcgcccttgctcaccattattaatttagtgtgtgtatttg	
CT-F2	cacaaatacacacactaaattaataatggtgagcaagggcgaggag	
CT-R2	gcctgttgctatcgataccgtcgacatagcgccgatcaaagtatttg	
CT-F3	tcggcgctatgtcgacggtatcgatagcaacaggcgcgttggac	
CT-R3	ctaaagggaacaaaagctggagctccaggaagaatacactatactg	
CF-F	cgctatgtcgac tgggtcattacgtaaataatgatag	p406-CF (ligation)
CF-R	ctcacgaattccat tttgaatatgtattacttggttatg	
CH-F	cgctatgtcgac gttttgacaccgagccatagc	p406-CH (ligation)
CH-R	ctcacgaattccat tattttattgtattgattgttg	
CR1-F	cgctatgtcgac catccacatattttaatcac	p406-CR1 (ligation)
CR1-R	ctcacgaattccat cgctggatatgcctagaaatg	
CR2-F	cgctatgtcgac aactatgcgaaatccggagcaac	p406-CR2 (ligation)
CR2-R	ctcacgaattccat ggtaattggacaaataaatac	
NmCas9-F	gttcgcggatcc atggtgcctaagaagaagagaaag	pH5-NmCas9 (ligation)
NmCas9-R	cacccgctcgag ttaatccagcttctttttcttcg	
St1Cas9-F	gttcgcggatcc atgagcgacctggtgctgggcctg	pH5-St1Cas9 (ligation)
St1Cas9-R	cacccgctcgag tcacaccttcctcttcttcttgg	
SaCas9-F	gacatgccatggggaaacggaactacatcctg	pH5-SaCas9 (ligation)
SaCas9-R	gaacgcgtcgacttacttgtcatcgtcatccttg	
AsCpf1-F	gttcgcggatcc atgacacagttcgagggctttac	pH5-As(Lb) Cpf1 (ligation)
LbCpf1-F	gttcgcggatcc atgagcaagctggagaagtttacaaactg	
Cpf1-R	cacccgctcgag tca ctttttctttttgcctggcc	
SgH-F	ccactacgtgctcgagtctttgaaaagataatg	pSgH (ligation)
SgH-R	Gcagggagctcagacataaaaaacaaaaaaa<br>ggagacctcggtctccgatcatttatctttcactgc	
SpSgH-F	ctccgcagtgaaagataaatgatcggagaccgaggtctccgttttagagctagaaatagc	pSpSgH (ligation)
SpSgH-R	cagacataaaaaacaaaaaaa ggatcaaaaaagcaccgactcggtg	
NmSgH-F	ctccgcagtgaaagataaatgatcggagaccgaggtctccgttgtagctccctttctcat	pNmSgH (ligation)
NmSgH-R	cagacataaaaaacaaaaaaa ggatctaaacgatgccccttaaagc	
St1SgH-F	ctccgcagtgaaagataaatgatcggagaccgaggtctccgtttttgtactctcagaaat	pSt1SgH (ligation)
St1SgH-R	cagacataaaaaacaaaaaaa ggatcaaaaaaacaccctgccataaaatg	
SaSgH-F	ctccgcagtgaaagataaatgatcggagaccgaggtctccgttttagtactctgtaattt	pSaSgH (ligation)
SaSgH-R	cagacataaaaaacaaaaaaa ggatcaaaaaaatctcgccaacaag	
NLS-BamHI-F	gatccatgcctccaaaaaagaagagaaaggtcggtagtggttctg	Insert N-terminal NLS at BamHI or NcoI site
NLS-BamHI-R	gatccagaaccactaccgacctttctcttcttttttggaggcatg	
NLS-NcoI-F	catgggccctccaaaaaagaagagaaaggtcggtagtggttcttc	
NLS-NcoI-R	catggaagaaccactaccgacctttctcttcttttttggagggcc	
ADE2-KO-F	atggattctagaacagttggtatattaggagggggacaatttcgtacgctgcaggtcgac	linear donor for *ADE2* deletion
ADE2-KO-R	ttacttgttttctagataagcttcgtaaccgacagtttctgcataggccactagtggatc	
Csy4-F	gttggaagatctatg ggtgatcattatctggatattc	pH3-Csy4 (ligation)
Csy4-R	cacccgctcgag tta aaaccagggcacgaaac	
dCas9-AD-F	actttttacaacaaatataaaacaGatggactacaaagaccatgacggtg	pH6-dSp/St1-Cas9-V/VP/VPR (Gibson)
dCas9-V-R	gaattaataaaagtgttcgcaaaggatctcacagcaaggctgagaaatccatatc	
dCas9-VP-R	gaattaataaaagtgttcgcaaaggatctcataacatatcgagatcgaaatc	
dCas9-VPR-R	gaattaataaaagtgttcgcaaaggatctcaagaagcgtagtccggaacgtc	

dSaCas9-AD-F	actttttacaacaaatataaaacagatggccccaaagaagaagcggaag	pH6-dSaCas9-V/VP/VPR (Gibson)
dSaCas9-V-R	gaattaataaaagtgttcgcaaaggatccagcatgtccaggtcgaaatcatcaag	
dSaCas9-VPR-R	gaattaataaaagtgttcgcaaaggatctcaaaacagagatgtgtcgaagatg	
dLbCpf1-F1	ccgccaccatggct cctccaaaaaagaagagaaag	dLbCpf1 OE-PCR
dLbCpf1-R1	caccacgatatacagcagattgcgctcgcccctagcgatgccgatcacataggggttatc	
dLbCpf1-F2	ctgaagcacgacgataacccctatgtgatcggcatcgctaggggcgagcgcaatctgctg	
dLbCpf1-R2	ccgccgaagcttctttttctttttgcctggccgg	
RD1-F	agttccaagcttggcggcagcggcggcagc atgactgccagcgtttcgaatac	Amplification of RD1/RD2/ RD3/RD4
RD1-R	cacccgctcgag tta aggtggttgctgttgttgaagttg	
RD2-F	agttccaagcttggcggcagcggcggcagc tacgaagaagagatcaagcac	
RD2-R	cacccgctcgag tta cgcaactggaacagatgcagatg	
RD3-F	agttccaagcttggcggcagcggcggcagc gctagtttgcaccaggatcac	
RD3-R	cacccgctcgag tta agatttgtgtaactcaacgtc	
RD5-F	agttccaagcttggcggcagcggcggcagc gattcacaagttcaagaactg	Amplification of RD5/RD6
RD5-R	cacccgctcgag tcagtccatgtgtgggaaggg	
RD6-F	agttccaagcttggcggcagcggcggcagc actagtggtacgaatttgcac	
RD6-R	Same as RD5-R	
RD7-F	agttccaagcttggcggcagcggcggcagc atggtaatcttcaaagaacg	Amplification of RD7/RD8/ RD9
RD7-R	cacccgctcgag tta gataagtggcggtaatattg	
RD8-F	Same as RD7-F	
RD8-R	cacccgctcgag tta agatttgttattttctgcaatttg	
RD9-F	agttccaagcttggcggcagcggcggcagc ttctgtcaagttttcgtaacaaag	
RD9-R	cacccgctcgag ttaaacttttaggccattgac	
RD10-F	agttccaagcttggcggcagcggcggcagc tgtgtagtgaacttgcaaaac	Amplification of RD10
RD10-R	cacccgctcgag tta atcacggaggtatctcaaccg	
RD11-F	agttccaagcttggcggcagcggcggcagc aattctgcatcttcatctac	Amplification of RD11
RD11-R	cacccgctcgag tta tgtagaattgttgctttcgaaaatg	
N-RD-F	ccgccaccatggct cccaagaaaaagcgcaaggtag	Insert RD2 and RD5 at N-terminus
N-RD2-R	gaggagccatggacgcaactggaacagatgcagatg	
N-RD5-R	gaggagccatggagtccatgtgtgggaagggcaacg	
3gRNA-F1	nnnnnggtctccggactctttgaaaagataatgtatg	Assemble three gRNA cassettes into a single plasmid using Golden-Gate Assembly
3gRNA-R1	nnnnnggtctcccggacttgcatgcctgcagggagctc	
3gRNA-F2	nnnnnggtctcctccgtctttgaaaagataatgtatg	
3gRNA-R2	nnnnnggtctcccctggcttgcatgcctgcagggagctc	
3gRNA-F3	nnnnnggtctccccagtctttgaaaagataatgtatg	
3gRNA-R3	nnnnnggtctccccaaccttgcatgcctgcagggagctc	
ReFu-F1	ggttgagtgttgttccagtttggaacaagagtc	Assemble CRISPR protein cassettes and Csy4 cassette into a single plasmid using DNA
ReFu-R1N	catgccggtagaggtgtggtcaataagag	
ReFu-F2N	agctttggacttcttcgccagaggtttg	
ReFu-R2N	gcttggtgccacttgtcacatacaattc	
ReFu-F3	cctgcagggtgtcgacgctgcgggtatagaaag	
ReFu-R3	ctgccctttatattccctgttacagcagccgagc	
ReFu-F4	gcggccgctatatctaggaacccatcaggttg	
ReFu-R4	gattgctatgctttctttctaatgagcaagaag	

ReFu-F5	ccgcggatagcttcaaaatgtttctactc	assembler
ReFu-R5	gggtttcgccacctctgacttgagcgtc	
SpMS2H-R	cagacataaaaaacaaaaaaa ggatc gggaagactccccagtgactg	pSpMS2SgH
SpPP7H-R	cagacataaaaaacaaaaaaa ggatc gggaactgctgcgtaagggtttc	pSpPP7SgH
SpComH-R	cagacataaaaaacaaaaaaa ggatc gatgctcgcaggcattcaggcaccgactcggtgc	pSpComSgH
PCP-MXI1-F1	gttcgcggatcc atgcccaaaaagaaaagaaaagtg	pH1-PCP-MXI1(ligation)
PCP-MXI1-R1	tcttgggagctccctc ggagccacggcccagcg	
MCP-VP64-F	gttggaagatct atgcccaaaaagaaaagaaaagtg	pH4-MCP-VP64 (ligation)
MCP-VP64-R	cacccgctcgag tcagttgatgagcatgtccagatc	
ROX1-Conf-F	tattctgttcagacagggacc	Verification and sequencing primers for CRISPRd
ROX1-Conf-R	gatagctgttcgagcttgacac	
PMR1-Conf-F	catctaacgaggccaacaatag	
PMR1-Conf-R	atataagctatacaagaggctg	
PEP4-conf-F	cgatcatgaagcttcatcaagc	
PEP4-conf-R	ctctccaattcggcgacttgac	
VPS8-conf-F	acgagaccggaaatatagagtg	
VPS8-conf-R	caggagaatggctagcggactg	
YPS1-conf-F	cgacttgaacgttaccgggttg	
YPS1-conf-R	tcagatggacagtccattgcgc	
qHMG1-F	agaagtggacggtgatttgag	Quantitative PCR analysis primers
qHMG1-R	catggcaccttgtggttcta	
qERG9-F	cttctggcccaaggaaatct	
qERG9-R	gacgaggtggtttatacagtcc	
qPDI1-F	gtcaacgacccaaagaagga	
qPDI1-R	tggcgtaggtatcagctagt	
qMNN9-F	ggagaaggaaagacacgcttta	
qMNN9-R	ccaagaagtgtgaggtcctatg	
qERO1-F	ttgctctgttgatgtcgtagag	
qERO1-R	tcatccgcttccttcattgtat	
qPMR1-F	ccttagcggttgctgctatt	
qPMR1-R	accttctcacgatggctttac	
qALG9-F	ccgttgccatgttgttgtatg	
qALG9-R	gccaggaaattgtacgctaaac	

Supplementary Table 9 | Oligos used to construct gRNAs.

Oligos	Sequences (5'-3')
pSg1-F	gatcttgatatttaagttattaaa
pSg1-R	aaactttaataacttaaatatcaa
pSg6-F	gatc actttagtgctgacacatac
pSg6-R	aaac gtatgtgtcagcactaaagt
pSg10-F	gatc gatatcaagaggattggaaa
pSg10-R	aaactttccaatcctcttgatatc
pSg12-F	gatc acgtccctattgaatgttgg
pSg12-R	caacccaacattcaatagggacgt
pSg13-F	gatc aactctggacattataccat
pSg13-R	caacatggtataatgtccagagtt
pSg14-F	gatc aaaaatgggcaccatttact
pSg14-R	aaacagtaaatggtgcccattttt
pSg15-F	gatc ccaattgtagagactatcca
pSg15-R	aaactggatagtctctacaattgg
pSg27-F	gatc tttaagttattaaa
pSg27-R	aaactttaataacttaaa
pSg28-F	gatc taaatatcaatggg
pSg28-R	aaac cccattgatattta
pSg29-F	gatc gaagctcatttgagatcaat
pSg29-R	caac attgatctcaaatgagcttc
pSg30-F	gatc ggaagaggtaacttcgttgt
pSg30-R	aaac acaacgaagttacctcttcc
pSg31-F	gatc gcaagcatcaatggtataatgtc
pSg31-R	aaacgacattataccattgatgcttgc
pSg32-F	gatcgtaacttcgttgtaaagaataagg
pSg32-R	caacccttattctttacaacgaagttac
pSg33-F	gatcgtgctgacacatac
pSg33-R	aaacgtatgtgtcagcac
pSg35-F	gatc gatatttaagttattaaa
pSg35-R	tttaataacttaaatatc
pSg36-F	gatc tatttaagttattaaa
pSg36-R	aaac tttaataacttaaata
pSg37-F	gatc atttaagttattaaa
pSg37-R	aaac tttaataacttaaat
pSg38-F	gatc ttaagttattaaa
pSg38-R	aaac tttaataacttaa
pSg39-F	gatc taagttattaaa
pSg39-R	aaac tttaataactta
pSg40-F	gatc agttattaaa
pSg40-R	aaac tttaataact
pSg55-F	gatc gaaaaaagtagcta
pSg55-R	aaac tagctacttttttc
pSg56-F	gatc ccgtaccataccct
pSg56-R	aaac agggtatggtacgg
pSg64-F	gatc ggatagtctctacaattggg
pSg64-R	aaac cccaattgtagagactatcc

pSg65-F	gatctccgccaggcgtgtatatatagc
pSg65-R	aaacgctatatatacacgcctggcgga
pSg66-F	gatcaacgaagcaggaaatgagagaat
pSg66-R	aaacattctctcatttcctgcttcgtt
pSg68-F	gatctaatttctactcttgtagatgatatcaagaggattggaaaagg
pSg68-R	aaaaccttttccaatcctcttgatatcatctacaagagtagaaatta
pSg69-F	gatcaatttctactaagtgtagatgatatcaagaggattggaaaagg
pSg69-R	aaaaccttttccaatcctcttgatatcatctacacttagtagaaatt
pSg87-F	gatccgaagcaggaaatgagagaat
pSg87-R	aaacattctctcatttcctgcttcg
pSg88-F	gatccttcgttcatttcgagtttcc
pSg88-R	aaacggaaactcgaaatgaacgaag
pSg89-F	gatccagacctccctgcgagcgggc
pSg89-R	aaacgcccgctcgcagggaggtctg
pSg90-F	gatccgccaggcgtgtatatatagc
pSg90-R	aaacgctatatatacacgcctggcg
pSg91-F	gatctcatttggcgagcgttggttg
pSg91-R	aaaccaaccaacgctcgccaaatga
pSg92-F	gatcgatctttccggtctctttggc
pSg92-R	aaacgccaaagagaccggaaagatc
pSg93-F	gatcggcttgttccacaggaacact
pSg93-R	aaacagtgttcctgtggaacaagcc
pSg94-F	gatcgccaaagtcctcgacttcaag
pSg94-R	aaaccttgaagtcgaggactttggc
pSg95-F	gatcacaacttcgccttaagttgaa
pSg95-R	aaacttcaacttaaggcgaagttgt
pSg109-F	gatctctaagttttaattacaaaa
pSg109-R	aaacttttgtaattaaaacttaga
pSg110-F	gatcggaattcgtgagcaagggcg
pSg110-R	aaaccgcccttgctcacgaattcc
pSg111-F	gatccgaggagctgttcaccgggg
pSg111-R	aaaccccggtgaacagctcctcg
pSg112-F	gatcgaccaggatgggcaccaccc
pSg112-R	aaacgggtggtgcccatcctggtc
pSg113-F	gatccgtcgccgtccagctcgacc
pSg113-R	aaacggtcgagctggacggcgacg
pSg114-F	gatcggtggtgcagatcagcttca
pSg114-R	aaactgaagctgatctgcaccacc
pSg115-F	gatccaagtaatacatattcaaaa
pSg115-R	aaacttttgaatatgtattacttg
pSg116-F	gatcgaatatgtattacttggtta
pSg116-R	aaactaaccaagtaatacatattc

pSg117-F	gatcaagaacagaagaataacgca
pSg117-R	aaactgcgttattcttctgttctt
pSg118-F	gatcttatccctcatgttgtctaa
pSg118-R	aaacttagacaacatgagggataa
pSg119-F	gatccaatcaatacaataaaataa
pSg119-R	aaacttattttattgtattgattg
pSg120-F	gatctactcttttgaacaagatgt
pSg120-R	aaacacatcttgttcaaaagagta
pSg121-F	gatcataagtatattaggatgagg
pSg121-R	aaaccctcatcctaatatacttat
pSg122-F	gatcaatttctactaagtgtagat gtgtaggaacatcaacatgctca
pSg122-R	aaaatgagcatgttgatgttcctacac atctacacttagtagaaatt
pSg123-F	gatcaatttctactaagtgtagat cccttctccagaaacaatcagat
pSg123-R	aaaaatctgattgtttctggagaaggg atctacacttagtagaaatt
pSg124-F	gatcaatttctactaagtgtagat ccattcgtcttgaagtcgaggac
pSg124-R	aaaagtcctcgacttcaagacgaatgg atctacacttagtagaaatt
pSg125-F	gatcaatttctactaagtgtagat agttattaaatggtcttcaattt
pSg125-R	aaaa aaattgaagaccatttaataact atctacacttagtagaaatt
pSg126-F	gatcaatttctactaagtgtagat ataacttaaatatcaatgggagg
pSg126-R	aaaa cctcccattgatatttaagttat atctacacttagtagaaatt
pSg127-F	gatcaatgaacgaagcaggaaatg
pSg127-R	aaaccatttcctgcttcgttcatt
pSg128-F	gatcgcgtgttgttgctgctgaca
pSg128-R	aaactgtcagcagcaacaacacgc
pSg135-F	gatcaatttctactaagtgtagat cttcttgctcattagaaagaaag
pSg135-R	aaaa ctttctttctaatgagcaagaag atctacacttagtagaaatt
pSg136-F	gatcaatttctactaagtgtagat taattaaaacttagattagattg
pSg136-R	aaaa caatctaatctaagttttaatta atctacacttagtagaaatt
pSg137-F	gatcaatttctactaagtgtagat cgtcgccgtccagctcgaccagg
pSg137-R	aaaa cctggtcgagctggacggcgacg atctacacttagtagaaatt
pSg138-F	gatctttcttagcaaagcaaagga
pSg138-R	aaactcctttgctttgctaagaaa
pSg139-F	gatcggaaactcgaaatgaacgaa
pSg139-R	aaacttcgttcatttcgagtttcc
pSg140-F	gatccttagcaaagcaaaggaggg
pSg140-R	aaaccccctcctttgctttgctaag
pSg141-F	gatcatagcggtagtgtttgcgcg
pSg141-R	aaaccgcgcaaacactaccgctat
pSg142-F	gatcgtaaaccccggccaaagaga
pSg142-R	aaactctctttggccggggtttac
pSg143-F	gatcacacgcctggcggatctgct
pSg143-R	aaacagcagatccgccaggcgtgt

pSg144-F	gatcacctgaatctaaaattcccg
pSg144-R	aaaccgggaattttagattcaggt
pSg146-F	gatcgccggggtttacggacgatg
pSg146-R	aaaccatcgtccgtaaaccccggc
pSg147-F	gatcgacggaaaaaagtagctaag
pSg147-R	aaaccttagctacttttttccgtc
pSg148-F	gatccaaagcattcaattcaaatg
pSg148-R	aaaccatttgaattgaatgctttg
pSg149-F	gatcaatttctactaagtgtagat caagggtatggtacggtgctatc
pSg149-R	aaaa gatagcaccgtaccatacccttg atctacacttagtagaaatt
pSg150-F	gatcaatttctactaagtgtagat tcagcagcaacaacacgctacgc
pSg150-R	aaaa gcgtagcgtgttgttgctgctga atctacacttagtagaaatt
Sg155-F	gatcaatttctactaagtgtagat cggacgatggcagaagaccaaag
Sg155-R	aaaa ctttggtcttctgccatcgtccg atctacacttagtagaaatt
Sg156-F	gatcaatttctactaagtgtagat gcgagcgttggttggtggatcaa
Sg156-R	aaaa ttgatccaccaaccaacgctcgc atctacacttagtagaaatt
Sg157-F	gatcaatttctactaagtgtagat gtgctgacacatacaggcatata
Sg157-R	aaaa tatatgcctgtatgtgtcagcac atctacacttagtagaaatt
pSg172-F	gatcataaatggaaagttaggaca
pSg172-R	aaactgtcctaactttccatttat
pSg175-F	gatcaatttctactaagtgtagatcggctatgaaaagctgttgttcg
pSg175-R	aaaacgaacaacagcttttcatagccgatctacacttagtagaaatt
pSg194-F	gatcaatttctactaagtgtagatcatattcgaagcttacaatcgag
pSg194-R	aaaactcgattgtaagcttcgaatatgatctacacttagtagaaatt
pSg195-F	gatcaatttctactaagtgtagattaccagcaatcagctgactaaca
pSg195-R	aaaatgttagtcagctgattgctggtaatctacacttagtagaaatt
pSg196-F	gatcaatttctactaagtgtagatttgctcttacccgactctgaaga
pSg196-R	aaaatcttcagagtcgggtaagagcaaatctacacttagtagaaatt
pSg197-F	gatcaatttctactaagtgtagatgcaagacctcaaacaatcgtact
pSg197-R	aaaaagtacgattgtttgaggtcttgcatctacacttagtagaaatt
pSg198-F	gatcgctggggtagaactagagta
pSg198-R	aaactactctagttctaccccagc
pSg199-F	gatcttatatgacagttcaaaaga
pSg199-R	aaactcttttgaactgtcatataa
pSg200-F	gatcggaagtggagatggaagagg
pSg200-R	aaaccctcttccatctccacttcc
pSg201-F	gatcctacatgcaaacgacaaata
pSg201-R	aaactatttgtcgtttgcatgtag
pSg202-F	gatcgctgaaaactgtatgtgcgg
pSg202-R	aaacccgcacatacagttttcagc
pSg203-F	gatcatccaacgatgcaattcagt
pSg203-R	aaacactgaattgcatcgttggat

pSg204-F	gatcaaatgggaatggaaagaacg
pSg204-R	aaaccgttctttccattcccattt
pSg217-F	gatcaatttctactaagtgtagatcaacaactatctgcgataactca
pSg217-R	aaaatgagttatcgcagatagttgttgatctacacttagtagaaatt
pSg218-F	gatcaatttctactaagtgtagatcagggtcttctataagagaaacc
pSg218-R	aaaaggtttctcttatagaagaccctgatctacacttagtagaaatt
pSg219-F	gatcaatttctactaagtgtagatagccctacttaatgctgagccac
pSg219-R	aaaagtggctcagcattaagtagggctatctacacttagtagaaatt
pSg220-F	gatcaatttctactaagtgtagatgctatgttagctgcaactttcta
pSg220-R	aaaatagaaagttgcagctaacatagcatctacacttagtagaaatt
pSg221-F	gatcaatttctactaagtgtagatgaaacacgtgtcctgaaaattat
pSg221-R	aaaaataattttcaggacacgtgtttcatctacacttagtagaaatt
pSg222-F	gatcaatttctactaagtgtagataaaatcatcgaatagccgatcga
pSg222-R	aaaatcgatcggctattcgatgattttatctacacttagtagaaatt
pSg223-F	gatcaatttctactaagtgtagatccagtcactatcatcatcatcat
pSg223-R	aaaaatgatgatgatgatagtgactggatctacacttagtagaaatt
pSg224-F	gatcaatttctactaagtgtagatacgggcaaaaactggattctccc
pSg224-R	aaaagggagaatccagtttttgcccgtatctacacttagtagaaatt
pSg225-F	gatcaatttctactaagtgtagattgtcttacgagccgggtaccaag
pSg225-R	aaaacttggtacccggctcgtaagacaatctacacttagtagaaatt
pSg226-F	gatcaatttctactaagtgtagatcaggggcgatgccacttatcagt
pSg226-R	aaaaactgataagtggcatcgcccctgatctacacttagtagaaatt
pSg227-F	gatcggattggcgagaaataatgt
pSg227-R	aaacacattatttctcgccaatcc
pSg228-F	gatcgcagatggggagagagaatg
pSg228-R	aaaccattctctctccccatctgc
pSg229-F	gatctttccttgtagcgatcaggt
pSg229-R	aaacacctgatcgctacaaggaaa
pSg230-F	gatcgaaataacgggtcccaagag
pSg230-R	aaacctcttgggacccgttatttc
pSg231-F	gatccccacgggttctttcttagg
pSg231-R	aaaccctaagaaagaacccgtggg
pSg260-F	gatcgcgagcaaacactattatga
pSg260-R	aaactcataatagtgtttgctcgc
pSg261-F	gatcagaagggcttggtttcgaaa
pSg261-R	aaactttcgaaaccaagcccttct
pSg262-F	gatccaaaacgggatatttaagcc
pSg262-R	aaacggcttaaatatcccgttttg
pSg263-F	gatcagccgaatgaatgaaatatg
pSg263-R	aaaccatatttcattcattcggct
pSg264-F	gatcttgttgtgatgatacaagag
pSg264-R	aaacctcttgtatcatcacaacaa

Supplementary Table 10 | gBLOCKs used in this study.

	Sequences
Sg10	ctttggtctccgatc<br>aaattctcctgccaaacaaataagcaactccaatgaccacgttaatggct<br>aatcctcttgatatcgaaaaactagctgaaaaatgtgatgtgctaacgat<br>gatatcaagaggattggaaa<br>gtttggagacctttc
Sg145	ctttggtctccgatc<br>tccacaaggacaatatttgtgacttatgttatgcgcctgctagagttccg<br>ggcagaaaatgcaatcaaatcttttcccggttgtggtatatttggtgtgg<br>acaacttcgccttaagttgaa<br>gtttggagacctttc
Sg163	gttcgcggatcc gttcactgccgtataggcagAATTTCTACTAAGTGTAGAT gcgagcgttggttggtggatcaa<br>gttcactgccgtataggcaggaccaggatgggcaccacccGTTTTAGAGCTAGAAATAGCAAGTTAAAA<br>TAAGGCTAGTCCGTTATCAACTTGAAAAAGTGGCACCGAGTCGGTGC<br>gttcactgccgtataggcagtccacaaggacaatatttgtgacttatgttatgcgcctgctagagttccgggcagaaaatgcaatcaaat<br>cttttcccggttgtggtatatttggtgtggacaacttcgccttaagttgaaGTTTTAGTACTCTGGAAACAGAATC<br>TACTAAAACAAGGCAAAATGCCGTGTTTATCTCGTCAACTTGTTGGCGAGA<br>gttcactgccgtataggcag ctcgagcgggtg
Sg186	ctttggtctccgatc<br>ctacacctaagattccaagacccaagaacgcatttattctgttcagacag<br>ggaccgctcaaggtgtggaaataccccataattcaaacatttctaaaatt<br>actaccacaggatcttaatag<br>gtttggagacctttc
Sg205	ctttggtctccgatc<br>tacataaaacctcacaaacgatcgaaaaatcttcctttaacgatcagcct<br>cttgtatagcttatatgggtacattagtcaaggaaggtcatggtaagggt<br>atctctcagaaatcggtacaa<br>gtttggagacctttc
Sg265	ctttggtctccgatc<br>cgatatcacttggttacctgttcgtcgtaaggcttactgggaagtcaagt<br>cgccgaattggagagccatggtgccgccatcgatactggtacttctttga<br>ttgaaggtatcggtttaggcg<br>gtttggagacctttc
Sg266	ctttggtctccgatc<br>catatgttccgatggtactcatgtagctgcctcataccagaccggaaata<br>tagagtgaaacccacttctgaaccaacaaatggtatgaccccaacgcctg<br>tatgcatttggaacttgaacg<br>gtttggagacctttc
Sg267	ctttggtctccgatc<br>ttgcgcacctagttcagtagcgatcatacttaccactgtttgaggtaaat<br>accaccaaaatcgaacactatttccatactatcatcagatggacagtcca<br>atacgtaataccctatcctgg<br>gtttggagacctttc

The gRNA targeting sequences were underlined, the gRNA scaffold sequences were shown in uppercase, and the Csy4 sites were shown in red.

Supplementary Table 11 | Yeast strains used in this study.

Strains	Genotype
CEN.PK2-1C	MAT*a*; *his3D1; leu2-3_112; ura3-52; trp1-289; MAL2-8c; SUC2*
CEN-iAID6	CEN.PK2-1C-KanMX-*TDH3p-dLbCpf1-VP64-p65-ADH1t-ENO2p-Csy4-PGK1t-TPI1p-dSpCas9-RD11-RD5-RD2-TPI1t-TEF1p-SaCas9-TEF1t*
CT	CEN.PK2-1C-*ura3::URA3-CYC1p-mCherry-TEF1t-TEF1p-mVenus-PGK1t*
CF	CEN.PK2-1C-*ura3::URA3-CYC1p-mCherry-TEF1t-FBA1p-mVenus-PGK1t*
CH	CEN.PK2-1C-*ura3::URA3-CYC1p-mCherry-TEF1t-HHF2p-mVenus-PGK1t*
CR1	CEN.PK2-1C-*ura3::URA3-CYC1p-mCherry-TEF1t-REV1p-mVenus-PGK1t*
CR2	CEN.PK2-1C-*ura3::URA3-CYC1p-mCherry-TEF1t-RNR2p-mVenus-PGK1t*
CEN-Crt	CEN-iAID6-*ura3::URA3-TDH3p-CrtYB-CYC1t-TDH3p-CrtE-CYC1t-TDH3p-CrtI-CYC1t*
CEN-EGII	CEN-iAID6-*ura3::URA3-TEF1p-prepro-HisTag-EGII-AGA1-PGK1t*

Supplementary References

1. Edmondson, D. G., Smith, M. M. & Roth, S. Y. Repression domain of the yeast global repressor Tup1 interacts directly with histones H3 and H4. *Genes Dev.* **10**, 1247-1259 (1996).
2. Ostling, J., Carlberg, M. & Ronne, H. Functional domains in the Mig1 repressor. *Mol. Cell. Biol.* **16**, 753-761 (1996).
3. Zhang, Z. & Reese, J. C. Molecular genetic analysis of the yeast repressor Rfx1/Crt1 reveals a novel two-step regulatory mechanism. *Mol. Cell. Biol.* **25**, 7399-7411 (2005).
4. Traven, A., Staresincic, L., Arneric, M. & Sopta, M. The yeast protein Xtc1 functions as a direct transcriptional repressor. *Nucleic Acids Res.* **30**, 2358-2364 (2002).
5. Kadosh, D. & Struhl, K. Repression by Ume6 involves recruitment of a complex containing Sin3 corepressor and Rpd3 histone deacetylase to target promoters. *Cell* **89**, 365-371 (1997).
6. Liu, L., Zhang, Y., Liu, Z., Petranovic, D. & Nielsen, J. Improving heterologous protein secretion at aerobic conditions by activating hypoxia-induced genes in *Saccharomyces cerevisiae*. *FEMS Yeast Res.* **15** (2015).
7. Zhang, B., Chang, A., Kjeldsen, T. B. & Arvan, P. Intracellular retention of newly synthesized insulin in yeast is caused by endoproteolytic processing in the Golgi complex. *J. Cell Biol.* **153**, 1187-1198 (2001).
8. Wentz, A. E. & Shusta, E. V. A novel high-throughput screen reveals yeast genes that increase secretion of heterologous proteins. *Appl. Environ. Microbiol.* **73**, 1189-1198 (2007).
9. Hou, J., Tyo, K. E., Liu, Z., Petranovic, D. & Nielsen, J. Metabolic engineering of recombinant protein secretion by *Saccharomyces cerevisiae*. *FEMS Yeast Res.* **12**, 491-510 (2012).
10. Valkonen, M., Penttila, M. & Saloheimo, M. Effects of inactivation and constitutive expression of the unfolded- protein response pathway on protein production in the yeast *Saccharomyces cerevisiae*. *Appl. Environ. Microbiol.* **69**, 2065-2072 (2003).
11. Becerra, M., Díaz Prado, S., Cerdán, E. & González Siso, M. I. Heterologous *Kluyveromyces lactis* β-galactosidase secretion by *Saccharomyces cerevisiae* super-secreting mutants. *Biotechnol. Lett.* **23**, 33-40 (2001).
12. Smith, J. D., Tang, B. C. & Robinson, A. S. Protein disulfide isomerase, but not binding protein, overexpression enhances secretion of a non-disulfide-bonded protein in yeast. *Biotechnol. Bioeng.* **85**, 340-350 (2004).
13. Tang, H. *et al.* N-hypermannose glycosylation disruption enhances recombinant protein production by regulating secretory pathway and cell wall integrity in *Saccharomyces cerevisiae*. *Sci. Rep.* **6**, 25654 (2016).
14. Hou, J., Tyo, K., Liu, Z., Petranovic, D. & Nielsen, J. Engineering of vesicle trafficking improves heterologous protein secretion in *Saccharomyces cerevisiae*. *Metab. Eng.* **14**, 120-127 (2012).
15. Larsson, S., Cassland, P. & Jonsson, L. J. Development of a *Saccharomyces cerevisiae* strain with enhanced resistance to phenolic fermentation inhibitors in lignocellulose hydrolysates by heterologous expression of laccase. *Appl. Environ. Microbiol.* **67**, 1163-1170 (2001).
16. Chen, Y., Pioli, D. & Piper, P. W. Overexpression of the gene for polyubiquitin in yeast confers increased secretion of a human leucocyte protease inhibitor. *Biotechnology (N Y)* **12**, 819-823 (1994).
17. Lian, J. & Zhao, H. Reversal of the β-oxidation cycle in *Saccharomyces cerevisiae* for production of fuels and chemicals. *ACS Synth. Biol.* **4**, 332-341 (2015).
18. Lian, J. & Zhao, H. Functional reconstitution of a pyruvate dehydrogenase in the cytosol of *Saccharomyces cerevisiae* through lipoylation machinery engineering. *ACS Synth. Biol.* **5**, 689-697 (2016).
19. Du, J., Yuan, Y., Si, T., Lian, J. & Zhao, H. Customized optimization of metabolic pathways by combinatorial transcriptional engineering. *Nucleic Acids Res.* **40**, e142 (2012).
20. Bao, Z., Jain, S., Jaroenpuntaruk, V. & Zhao, H. Orthogonal genetic regulation in human cells using chemically induced CRISPR/Cas9 activators. *ACS Synth. Biol.* (2017).

21. Chavez, A. *et al.* Highly efficient Cas9-mediated transcriptional programming. *Nat. Methods* **12**, 326-328 (2015).
22. Kiani, S. *et al.* Cas9 gRNA engineering for genome editing, activation and repression. *Nat. Methods* **12**, 1051-1054 (2015).
23. Bao, Z. *et al.* Homology-integrated CRISPR-Cas (HI-CRISPR) system for one-step multigene disruption in *Saccharomyces cerevisiae*. *ACS Synth. Biol.* **4**, 585-594 (2015).
24. Gilbert, L. A. *et al.* CRISPR-mediated modular RNA-guided regulation of transcription in eukaryotes. *Cell* **154**, 442-451 (2013).
25. Hou, Z. *et al.* Efficient genome engineering in human pluripotent stem cells using Cas9 from *Neisseria meningitidis*. *Proc. Natl. Acad. Sci. U. S. A.* **110**, 15644-15649 (2013).
26. Kleinstiver, B. P. *et al.* Engineered CRISPR-Cas9 nucleases with altered PAM specificities. *Nature* **523**, 481-485 (2015).
27. Zetsche, B. *et al.* Cpf1 is a single RNA-guided endonuclease of a class 2 CRISPR-Cas system. *Cell* **163**, 759-771 (2015).
28. Zalatan, J. G. *et al.* Engineering complex synthetic transcriptional programs with CRISPR RNA scaffolds. *Cell* **160**, 339-350 (2015).
29. Verwaal, R. *et al.* High-level production of beta-carotene in *Saccharomyces cerevisiae* by successive transformation with carotenogenic genes from *Xanthophyllomyces dendrorhous*. *Appl. Environ. Microbiol.* **73**, 4342-4350 (2007).
